# Construction and validation of a transient receptor potential-related long noncoding RNA signature for prognosis prediction in breast cancer patients

**DOI:** 10.1097/MD.0000000000035978

**Published:** 2023-11-17

**Authors:** Qiaonan Guo, Pengjun Qiu, Kelun Pan, Jianpeng Chen, Baiwei Wang, Jianqing Lin

**Affiliations:** a Department of Breast and Thyroid Surgery, The Second Affiliated Hospital of Fujian Medical University, Quanzhou, China.

**Keywords:** breast cancer (BC), immune cell infiltration, long noncoding RNA (lncRNA), risk model, transient receptor potential (TRP) channels

## Abstract

Breast cancer (BC) is the most commonly diagnosed malignancy in women around the world. Accumulating evidence suggests that transient receptor potential (TRP) channels play a significant role in tumor progression and immune cell infiltration. Hence, we conducted the study to investigate the correlation between TRP-associated lncRNAs and the prognosis of breast carcinoma. In the current study, 33 TRP-associated genes were selected from a review published by Amrita Samanta et al, and the TRP-related lncRNAs were identified by Pearson analysis. Based on the sum of the expression levels of 12 lncRNAs provided by the Cancer Genome Atlas (TCGA), a TRP-associated lncRNA signature was established by using Cox regression analysis. According to the median value of the risk score in the training set, BC patients were separated into high- and low-risk groups. Subsequently, functional enrichment analysis was conducted on the differential expression genes (DEGs) between different risk groups. The Estimation of Stromal and Immune Cells in Malignant Tumor Tissues Using Expression (ESTIMATE) Score was calculated by ESTIMATE, and the immune cell infiltration was evaluated by ssGSEA. Finally, the immune checkpoint gene expression levels, microsatellite instability (MSI), and immunophenoscore (IPS) were further assessed. The high-risk groups exhibited lower survival rates, while the low-risk groups showed higher survival rates. As a result, the DEGs between different risk groups were highly enriched in immune cell activation and immunoregulation. Besides, the ESTIMATE scores of patients in low-risk groups were higher than those in high-risk groups. The infiltration levels of several immune cells were remarkably elevated in low-risk groups, and various immune signatures were activated with a decreased risk score. Eventually, the TRP-associated lncRNA signature was confirmed with a highly potential ability to evaluate the immunotherapy response in breast carcinoma patients. The outcomes of the current study indicated that the 12-TRP-associated-lncRNA risk model was an independent prognostic risk factor for BC patients. This risk model could be closely related to the tumor immune microenvironment in BC. Our findings will provide new insights for future immunotherapy for BC treatment.

## 1. Introduction

Breast cancer is one of the leading causes of cancer death in female around the word.^[1]^ As the pivotal health threat among women, breast tumor accounts for approximately 24.2% of all female malignant tumor cases and the account of all female carcinoma deaths is nearly 15%.^[2]^ As a result of improved diagnosis and treatment, the mortality rate from breast cancer has decreased year by year. However, breast carcinoma is a kind of highly heterogeneous malignant tumor, the treatment therapies and the response rates vary among different molecular subtypes. Despite of several treatment therapies, for instance surgery, chemotherapy, radiotherapy, targeted therapy and immunotherapy, patients with metastatic tumors still have clinical outcomes. Tumor metastasis and drug resistance are main causes of death in BC patients. Therefore, it is significant to screen novel reliable prognostic markers and therapeutic targets to achieve the individual precision treatment.

In recent years, immunotherapy has received increasing attention from academics. As significant regulators for immune system, lncRNAs perform various roles in specific stages of cancer immunity, such as antigen presentation, immune cell activation, and immune responses.^[3–5]^ Besides, lncRNAs were reported as potential prognostic biomarkers for breast cancer, which played an important role in tumor diagnosis and treatment.^[6]^

The development of tumors is related to variations in the cell cycle that inhibit pathways which cause cell death and shift the balance towards increased proliferation.^[7]^ Oftentimes, these variations are correlated with alterations in [Ca2+]i homeostasis in cells.^[8]^ TRP channels are identified as a group of cation channel proteins that act as signal transducers by altering membrane potential or intracellular Ca2 + concentration.^[9]^ TRP channels perform important roles in the rapid perception of external stimuli as well as in cell proliferation,^[10]^ differentiation,^[11]^ apoptosis,^[12]^ and drug resistance.^[13]^ Aberrant expression or activity of these channels may lead to serious disorders in humans. Based on the differences in sequence homology and topology, the TRP superfamilies can be divided into 7 common subfamilies: TRPC (canonical), TRPV (vanilloid), TRPM (melastatin), TRPA (ankyrin), TRPP (polycystin), TRPML (mucolipin) and TRPN (Drosophila NOMPC),^[14]^ and the subsequent discovery of an eighth TRP subfamily in yeast, named TRPY (Yeast).^[15]^ Interestingly, several studies previously indicated that TRP channels were closely associated with tumor progression and their roles varied according to different subfamilies. TRPC1 was identified as the first TRP channel in mammals,^[16]^ which was found to play different roles in specific types of carcinomas, for example, prostate cancer,^[9]^ nasopharyngeal carcinoma,^[17]^ and malignant glioma.^[18]^ A recent study in MCF-7 BC cell lines revealed that TRPV1 agonists and antagonists could inhibit the growth of cells.^[19]^ In addition, Zhou K et al suggested that overexpression of TRPV2 was related to poor prognosis of patients with esophageal squamous cell carcinoma.^[20]^ Also, a member of the TRPV subfamily, TRPV6 has been reported to be increased in estrogen receptor-negative BC and its inhibition reduced basal calcium influx and tumor cell growth.^[21]^ Notably, the TRPM family has been found to be involved in tumorigenesis, proliferation, and differentiation.^[22]^ TRPM1 was observed to be highly expressed in non-metastatic melanoma and due to this correlation, TRPM1 was considered as tumor suppressors and a potential prognostic marker for metastatic melanoma.^[23,24]^ Hopkins MM and colleagues investigated the role of TRPM2 in breast carcinoma progression and found that TRPM2 inhibition leaded to an increasing of DNA damage and decreasing of tumor proliferation.^[25]^ Accordingly, TRP channels may have a critical effect in the development and progression of malignant tumors. Despite the fact that these channels were commonly expressed in a wide range of tumors, their expression status, activities, and functions in BC remained poorly understood. Hence, the bioinformatic analysis was employed to investigate the relationship between TRP channels and the prognosis of patients with BC.

In addition to tumor progression, TRP channels were found to play an important role in the immune responses. Within the immune system, TRP channels were involved in a diverse range of functions including T and B cells activation, bactericidal activities of neutrophils and macrophages, antigen presentation by dendritic cells (DCs), and degranulation of mast cells.^[26]^ Studies in murine models of type 1 diabetes and multiple sclerosis have suggested the immunosuppressive role for TRPC5.^[27]^ Besides, TRPM2 channels were identified to be engaged in Ca2 + signaling within the granules of cytolytic natural killer (NK) cells.^[28]^ TRPM2 was activated after the malignant cells had been identified, leading to the granule polarization and degranulation mediated by Ca2+.^[26,28]^ Several studies revealed that TRP channel families were closely associated with the immune cells and immune responses regulation. However, there are seldom studies paying attention to the relationship between tumor immune microenvironment (TIME) and the expression status of TRP channels. Therefore, we conducted this study to investigate the role of TRP channels in TIME for breast cancer.

In this study, the BC lncRNA expression dataset and corresponding information from the cancer genome atlas (TCGA) were downloaded and analyzed. Subsequently, the prognostic lncRNAs associated with TRP channels were selected to establish a 12 TRP-related-lncRNA signature to further predict the survival prognosis of breast cancer patients. According to the TRP-related risk score, the differences in the infiltration rate of immune cells and functional enrichment analysis were investigated. After that, the relationship of TRP-related lncRNA risk score and TIME was searched. Furthermore, the role of TRP-related lncRNA risk score in the assessment of immunotherapy efficacy was explored. We aimed to investigate the association between TRP-related lncRNAs and the immune microenvironment of breast cancer. In the field, this pioneering therapeutic approach will be applied in the future to develop novel treatment combinations.

## 2. Methods and Materials

### 2.1. Workflow

**Figure 1.**
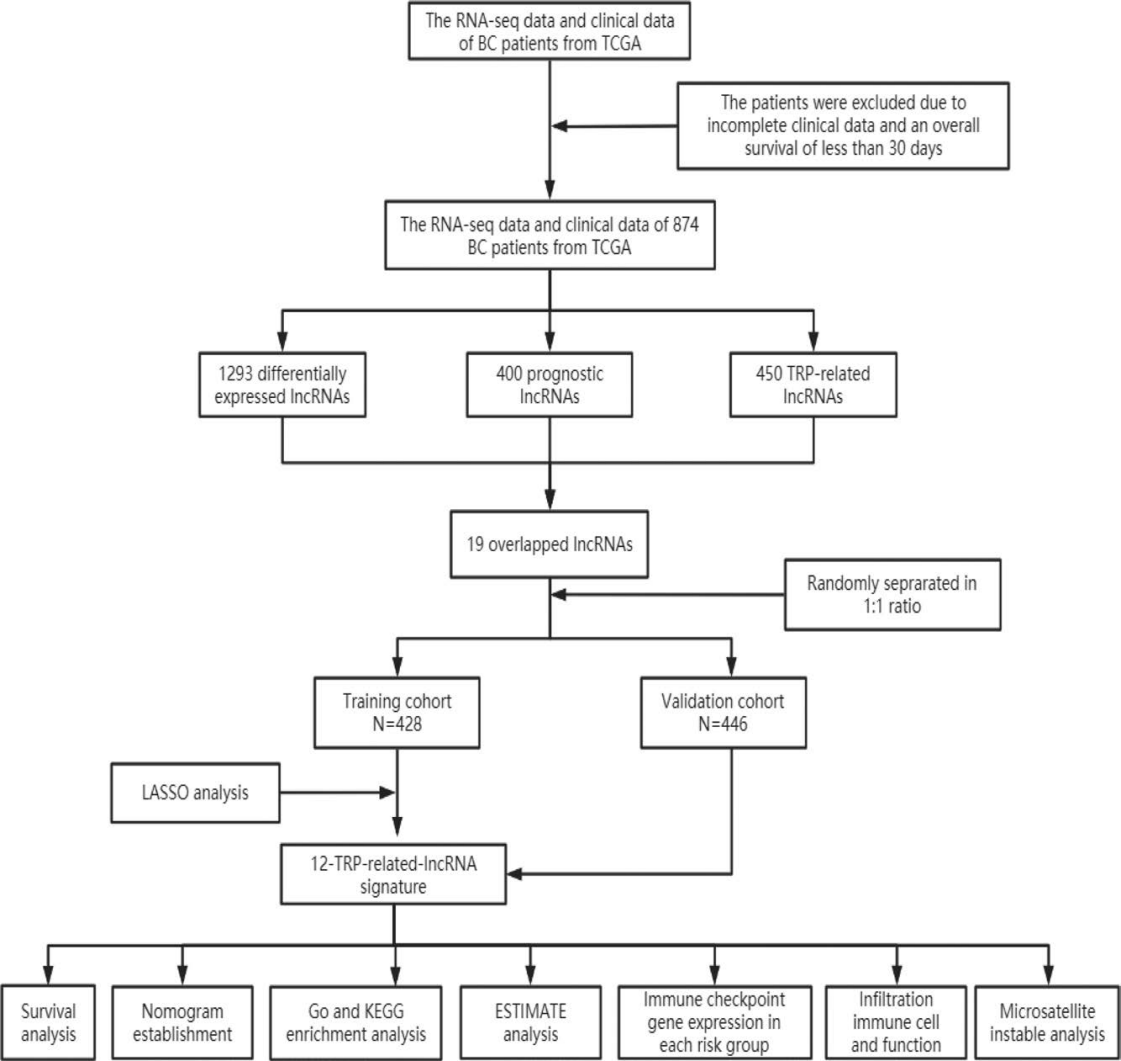
Analysis flow chart.

A sequential approach of a few steps was adopted to create a 12-TRP-related-lncRNA risk model and to explore the potential mechanisms by which these TRP-related lncRNAs affect BC prognosis (Fig. 1).

### 2.2. Data acquisition

The high-throughput sequencing-counts data of Transcriptome Profiling containing mRNA, lncRNA, and miRNA as well as corresponding information of BC patients were obtained from TCGA (http://cancergenome.nih.gov/) database. The conditions of eligible samples were as follows: (1) samples have both transcriptome expression data and completed clinical prognostic information; (2) samples excised from primary tumors were selected based on the naming principle. The exclusion criteria for samples were as follows: (1) samples with incomplete clinical data; (2) sample with an overall survival (OS) time of <30 days. As a result, 874 BC patients from the TCGA with complete follow up information were enrolled for subsequent analysis. 33 TRP-associated genes were selected from a review published by Amrita Samanta et al^[24]^ and provided in Supplementary Table 1, http://links.lww.com/MD/K642. The data from TCGA is publicly available, and the current research followed the TCGA data access policies and publication guidelines.

### 2.3. Selection of TRP-associated lncRNAs

According to the Ensemble database for human gene ID annotation information, expression matrices of lncRNAs and mRNAs were separated. In current study, after filtering out lncRNAs with a mean expression value of <1, 3158 lncRNAs were included for further analysis. Followed by Pearson correlation coefficients were computed to determine the correlation of TRP-related genes and the corresponding lncRNAs. Subsequently, the TRP-associated lncRNAs were screened out according to the standard that P (adjusted P) < 0.01 and |R| > 0.4.

### 2.4. Creation of a prognostic TRP-associated lncRNAs risk model

The “edge R” package of R software (R, A Language and Environment for Statistical Computing, R Core Team, R Foundation for Statistical Computing, Vienna, Austria. 2021, URL: http://www.R-project.org, R version 4.1.0) was employed to identify the differentially expressed lncRNAs between normal mammary tissues and tumor tissues. The differentially expressed lncRNAs were identified as meeting cutoff criteria of |log2fold change (FC)|> 1 and P (adjusted P) < 0.05, presented through volcano plot. After that, prognosis related lncRNAs were obtained by performing univariate Cox regression on OS. Subsequently, the Venn diagram was drawn to identify the candidate lncRNAs from differentially expressed lncRNAs, TRP-associated lncRNAs and prognostic lncRNAs. After that, there were 874 patients randomized in a 1:1 ratio to either a training set or a validation set to establish and validate the risk model of lncRNAs associated with TRP. Furthermore, the least absolute shrinkage and selection operator (LASSO) Cox regression model was constructed in training cohort to remove redundant lncRNAs and avoid model overfitting. Consequently, 12 independent prognostic TRP-associated lncRNAs were determined for the construction of risk model. The individual risk score of this prognostic feature was calculated based on the normalized expression levels of TRP-related lncRNAs and the relevant regression coefficients. The calculation formula is as follows: Risk score = $\overset{n}{\underset{i=1}{\sum }}\left(Exp_{i}*Coe_{i}\right)$. (N = 12, $Exp_{i}$ denotes the expression level of every TPR-associated lncRNA, and $Coe_{i}$ denotes the relevant Cox regression coefficient.) Accordingly, patients in the training queue were grouped into high-risk and low-risk cohorts on the basis of the median risk score. Afterward, the “survminer” R package was adopted to conduct the survival analysis on the high-risk and low-risk groups. Following by the time-dependent receiver operating characteristic (ROC) curve analysis was performed to evaluate the efficacy of this risk model for assessing the prognosis of BC patients. Subsequently, in the validation set, the individual risk score was calculated based on the same formula and the patients were grouped into high-risk and low-risk cohorts according to the same cutoff score as the training set. Similarly, the survival analysis and the time-dependent ROC curve analysis were performed in the validation set. Finally, the mentioned analyses were also conducted in the whole cohort and 5 random test validation cohorts.

### 2.5. Identification of the independent prognostic factors

The risk score based on the TRP-related lncRNAs signature and other clinical factors from the TCGA dataset were enrolled in univariate Cox regression and multivariate Cox regression to further identify the independent factor associated with BC patients’ prognosis.

### 2.6. Construction of a prognostic nomogram based on the risk score and clinical parameters

The nomogram was established from the 5-, 7-, and 10-year OS information of BC patients as well as the independent prognostic factors via the “rms” R package. The area under curve (AUC), and calibration plots were employed to assess the accuracy of the nomogram in 5, 7, and 10 years.

### 2.7. Construction of the lncRNA-mRNA co-expression network and functional enrichment analysis

The mRNA-lncRNA co-expression network was constructed by use of Cytoscape software to identify the correlation of TRP-associated lncRNAs and corresponding mRNAs and visualized through the Sankey diagram (|R| > 0.4 and *P* < .01). Gene Ontology (GO) enrichment analysis and Kyoto Encyclopedia of Genes and Genomes (KEGG)^[29–31]^ pathway analysis were conducted in the DEGs of different risk groups by use of the R clusterProfiler package (v3.10.1; https://www.bioconductor.org/packages/release/bioc/html/clusterProfiler.html).

### 2.8. Relevance assessment of risk score and TIME

Estimation of Stromal and Immune cells in Malignant Tumor tissues using expression (ESTIMATE) algorithm was adopted to obtain the proportion of the immune-stromal composition in TIME via “estimate” R package (https://bioinformatics.mdanderson.org/estimate/rpackage.html), for instance Stromal Score, Immune Score, and ESTIMATE Score. The corresponding scores indicated the ratio of the respective composition in the TIME. Furthermore, the “GSEAbase” R package (v1.58.0; https://www.bioconductor.org/packages/release/bioc/html/GSEABase.html) was employed to perform the single-sample gene set enrichment analysis to illustrate the enrichment of genomes associated with immune function.

### 2.9. Prediction of immunotherapy response

In accordance with the reported studies, the expression levels of genes associated with immune checkpoint was closely correlated with the efficacy of immune checkpoint inhibitors. Therefore, the ggplot2, ggpubr and ggExtra R packages (v1.13.0; https://www.bioconductor.org/packages/devel/bioc/html/DiscoRhythm.html) were employed to elucidate the correlation between the TRP-associated lncRNAs risk model and the expression of immune checkpoint blockade (ICB) related genes, including PD1, CD274, LAG3, CTLA4, and TIM3. Besides, the transcription expression of vital mismatch repair genes was counted and analyzed in breast carcinoma samples, such as MSH2, MSH6, MLH1, and PMS2. Additionally, the individual immunophenoscore (IPS) for samples was calculated by use of The Cancer Immunome Atlas database (https://tcia.at/), that could predict the response to CTLA-4 and PD-1. Eventually, the correlation between TPR-related risk scores and IPS was determined by comparing IPS in the high-risk and low-risk groups. Subsequently, the R package “maftools” (v2.13.0; https://www.bioconductor.org/packages/devel/bioc/html/maftools.html) was applied to calculate the tumor mutation burden, defined as the number of somatic mutations per-mega base of the genomic sequence being interrogated. *P* < .05 was regarded as statistically significant.

### 2.10. Statistical analysis

All statistical data were performed by R software (Version 4.1.0) (https://www.r-project.org/.). The relevance between TRP-associated genes and corresponding lncRNAs was identified by Pearson correlation analysis. The categorical variables were analyzed by Chi-square or fisher test, whereas the continuous data were analyzed by Wilcoxon test. The survival data were assessed with Kaplan–Meier curve and the independent prognostic factors were estimated via univariate and multivariate Cox regression analyses. *P* < .05 was considered statistically significant. All methods were carried out in accordance with relevant guidelines and regulations.

## 3. Results

### 3.1. Acquisition of TRP-related prognostic differentially expressed lncRNAs

**Figure 2.**
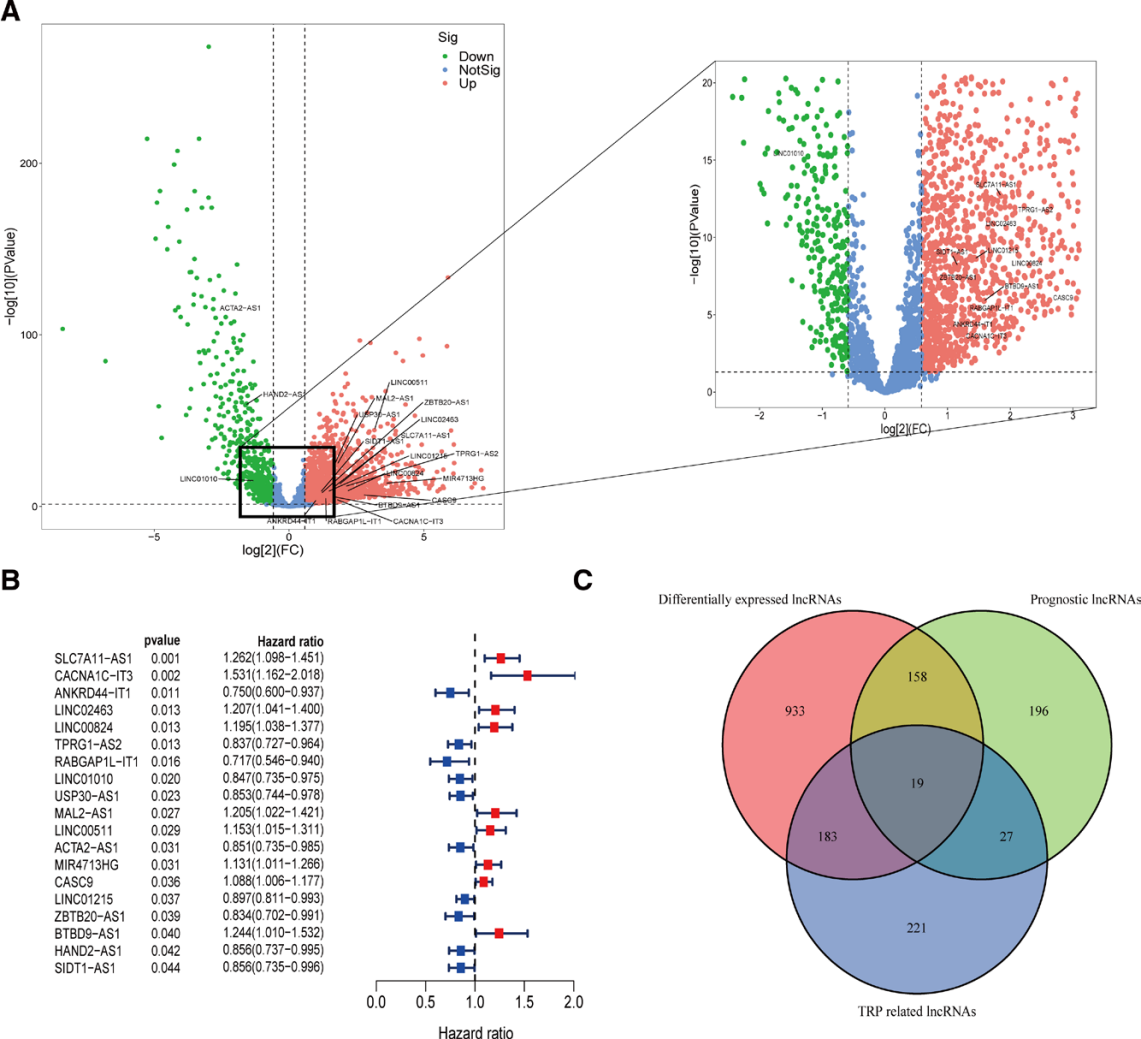
Identification of prognostic differential expression TRP-associated lncRNAs in BC patients. (A) Volcano plot of differential expression lncRNAs. Upregulated lncRNAs were shown by red spots and the downregulated ones were shown by green spots. (B) The forest plot showed the HR (95%CI) and *P* value of selected prognostic lncRNAs by univariate Cox proportional-hazards analysis. (C) The 19 overlapped lncRNAs of differential expression lncRNAs, prognostic lncRNAs and TRP-associated lncRNAs were shown by Venn diagram. BC = breast cancer, lncRNA = long noncoding RNA, TRP = transient receptor potential.

A total of 3158 lncRNAs were initially collected from 112 normal breast tissues and 874 breast tumor tissues form TCGA datasets through RNA-seq data analysis. Besides, the corresponding clinical information of patients in training and validation sets were shown in Table 1. 33 TRP-related genes were collected from previous publication, of which 30 genes were identified in the data from TCGA. After that, 450 lncRNAs were identified correlated (|R| > 0.4 and *P* < .01) with TRP-associated genes (Supplementary Table 2, http://links.lww.com/MD/K643). Subsequently, 1293 differentially expressed lncRNAs were screened out between tumor and normal tissues, of which 971 were up-regulated and 322 were down-regulated, visualized by Volcano map (Fig. 2A). Besides, 400 prognoses related lncRNAs were identified through univariate Cox regression analysis (Supplementary Table 3, http://links.lww.com/MD/K644), of which 19 final selected overlapping lncRNAs were presented on Figure 2B. Consequently, based on the all samples, the Venn diagram was drawn to identify the 19 overlapping lncRNAs from TRP-associated lncRNAs, differentially expressed lncRNAs, and prognostic lncRNAs as the candidate lncRNAs for further study (Fig. 2C).

**Table 1:** Clinicopathological features of patients with breast cancer in this study.

Variables	Training cohort (n = 428)		Validation cohort (n = 446)		*P* value
	No.	%	No.	%	
Survival	_-_	_-_	_-_	_-_	.834
Alive	385	90.0	398	89.2	_-_
Dead	43	10.0	48	10.8	_-_
Age	-	-	-	-	1
<=60	238	55.6	249	55.8	_-_
>60	190	44.4	197	44.2	_-_
T	_-_	_-_	_-_	_-_	.849
T1	123	28.7	119	26.7	_-_
T2	235	54.9	255	57.2	_-_
T3	56	13.1	60	13.5	_-_
T4	14	3.3	12	2.7	_-_
N	_-_	_-_	_-_	_-_	.383
N0	198	46.3	204	45.7	_-_
N1	158	36.9	148	33.2	_-_
N2	43	10.0	51	11.4	_-_
N3	27	6.3	37	8.3	_-_
Nx	2	0.5	6	1.3	_-_
M	_-_	_-_	_-_	_-_	.392
M0	352	82.2	375	84.1	_-_
M1	7	1.6	11	2.5	_-_
Mx	69	16.1	60	13.5	_-_
Stage	-	-	-	-	.573
Stage I	85	19.8	75	16.8	-
Stage II	238	56.4	257	57.6	-
Stage III	98	21.3	103	23. 1	-
Stage IV	7	2.5	11	2.5	-

### 3.2. Establishment of the TRP-related lncRNAs risk model

**Figure 3.**
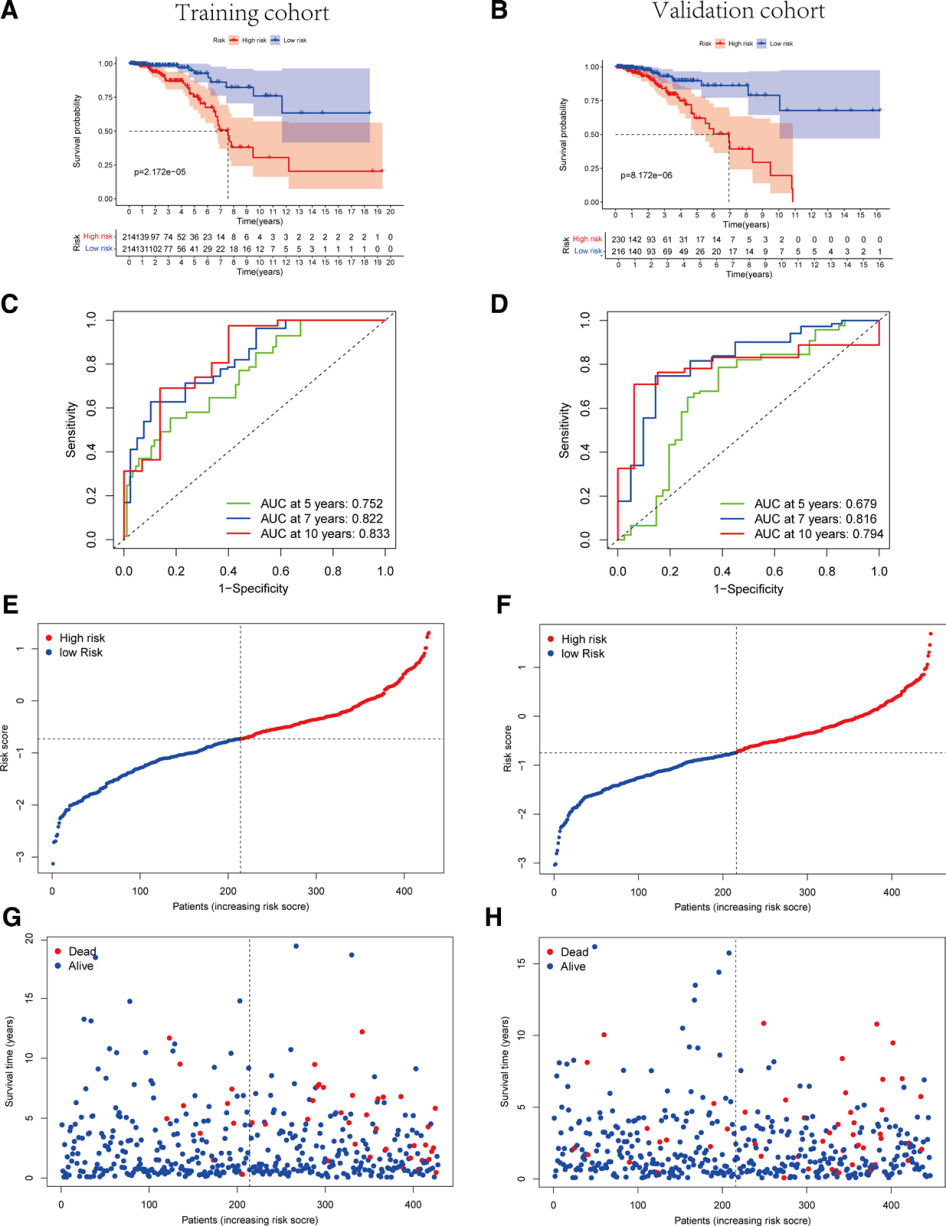
Assessment the predictive effectiveness of TRP-associated lncRNAs risk model in training cohort and validation cohort. (A, B) Kaplan–Meier survival curves for BC patients in training cohort (A) and validation cohort (B), indicated that the OS of the low-risk groups was higher than that of the high-risk groups, respectively (*P* = 2.172E-05 and *P* = 8.172E-06, respectively). (C, D) ROC curve analysis for the accuracy of the signature to predict prognosis of BC patients at 5, 7 and 10 years in the training (C) and validation (D) cohorts. The distribution and median value of the risk score in training cohort (E). The distribution and of the risk score in validation cohort (F) and the cutoff value of high and low risk sets was set as the median score of training cohort. The distributions of survival time status, and risk scores in training (G) and validation (H) cohorts (Fisher exact test: in training cohort, *P* = .0003083; in validation cohort, *P* = .0006565). BC = breast cancer, OS = overall survival, TRP = transient receptor potential.

LASSO regression analysis was performed on the 19 overlapping lncRNAs to prevent overfitting of the prediction model. The LASSO coefficient profiles of the 19 lncRNAs were provided (Supplementary Fig. 1A, http://links.lww.com/MD/K645) and 5-fold cross-validation results were generated to confirm the optimal values of the penalty parameter λ (λ = 0.009930509) (Supplementary Fig. 1B, http://links.lww.com/MD/K645). As a result, a total of 12 lncRNAs were selected for follow study: HAND2-AS1, LINC00511, MAL2-AS1, USP30-AS1, MIR4713HG, SLC7A11-AS1, TPRG1-AS2, LINC02463, SIDT1-AS1, BTBD9-AS1, RABGAP1L-IT1, and ANKRD44-IT1. Accordingly, TRP-related-lncRNA risk model to predict the clinical outcomes of BC sufferers was established on the basis of the expression of the 12 core lncRNAs and their regression coefficients as follows: Risk score = (-0.145 × expression level of HAND2-AS1) + (0.029 × expression level of LINC00511) + (0.193 × expression level of MAL2-AS1) + (-0.114 × expression level of USP30-AS1) + (0.067 × expression level of MIR4713HG) + (0.179 × expression level of SLC7A11-AS1) + (-0.146 × expression level of TPRG1-AS2) + (0.190 × expression level of LINC02463) + (-0.209 × expression level of SIDT1-AS1) + (0.149 × expression level of BTBD9-AS1) + (-0.120 × expression level of RABGAP1L-IT1) + (-0.288 × expression level of ANKRD44-IT1). The median risk score of training set was set as the cutoff value of high-risk group and low-risk group both in training and validation sets (Fig. 3E and F).

In training set, the survival analysis indicated worse OS of high-risk group patients. As shown by the Kaplan–Meier curves, the median survival time of patients in high-risk group was poor than that in low-risk group (*P* < .01) (Fig. 3A). Subsequently, a time-dependent ROC analysis revealed that the prognostic signature identified were confirmed promising efficient to be a prognosis predictor for breast carcinoma via the AUC (AUC = 0.752, 0.822 and 0.833; at 5, 7 and 10 years, respectively, Fig. 3C). As shown in Figure 3G, patients in high-risk group had a higher probability of death than those in low-risk group.

Similarly, 446 patients were included in validation cohort and the risk score of each patient was calculated according to the mentioned 12-TRP-related-lncRNA risk model. In validation set, patients in high-risk group were identified worse OS. As shown by the Kaplan–Meier curves, the median survival time of patients in high-risk group was poor than that in low-risk group (*P* < .01) (Fig. 3B). After that, the time-dependent ROC analysis was performed to verify the robust predictive efficiency of the risk model (AUC = 0.679, 0.816 and 0.794; at 5, 7 and 10 years, respectively, Fig. 3D). Notably, patients in high-risk group were manifested a higher probability of death than those in low-risk group (Fig. 3H).

After that, the risk score of each patient in the whole dataset was calculated and the survival analysis was performed. In the whole dataset, patients in high-risk group were identified worse OS and a higher probability of death than those in low-risk group. Also, the time-dependent ROC analysis was performed to confirm the predictive efficiency of the risk model (AUC = 0.717, 0.813 and 0.826; at 5, 7 and 10 years, respectively, Supplementary Fig. 2, http://links.lww.com/MD/K646).

Eventually, we have done 5 random selections from TCGA data to validate the risk model. According to all 5 test groups, patients in high-risk groups were identified worse OS than those in low-risk groups. The results indicated an excellent predictive performance of this risk model (Supplementary Fig. 3, http://links.lww.com/MD/K647).

### 3.3. Identification of independent prognostic predictors

**Figure 4.**
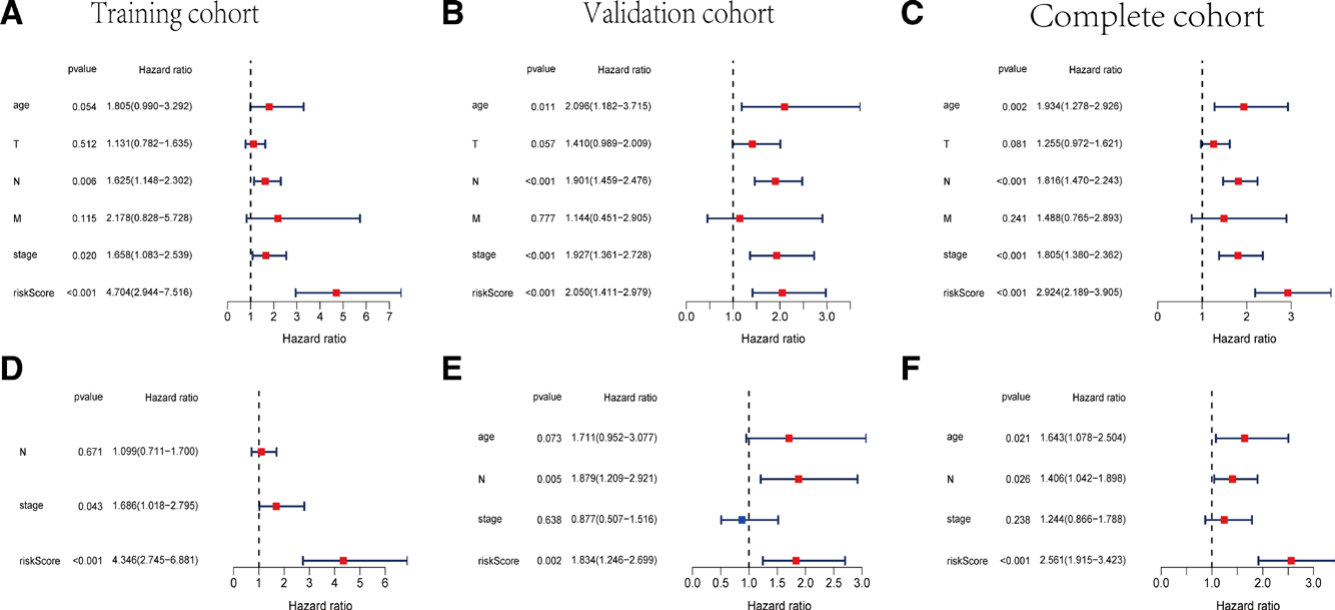
Identification of TRP-related lncRNAs risk model as an independent prognostic factor in BC. Univariate and multivariate Cox regression analyses of the risk model in the training cohort (A and D), validation cohort (B and E) and complete cohort (C and F). BC = breast cancer, lncRNA = long noncoding RNA, TRP = transient receptor potential.

In order to investigate the independent prognostic factors for breast cancer patients, the univariate and multivariate Cox regression analysis were conducted in the training, validation, and complete cohorts, respectively. The univariate Cox regression analysis indicated that the 12-TRP-related-lncRNA signature can play as an independent predictor for BC patients in training set (*P* < .001, HR = 4.704, 95% CI: 2.944–7.516, Fig. 4A) and validation set (*P* < .001, HR = 2.050, 95% CI: 1.411–2.979, Fig. 4B) Additionally, the multivariate Cox regression analysis revealed that the risk score was an independent variable for predicting the prognosis of patients in training set (*P* < .001, HR = 4.346, 95% CI: 2.745–6.881, Fig. 4D) and validation set (*P* = .002, H = 1.834, 95% CI: 1.246–2.699, Fig. 4E). Besides, in complete cohort, the univariate Cox regression analysis suggested that age, N status, tumor stage and risk score had the potential to predict the OS of BC patients (*P* = .002, HR = 1.934, 95% CI: 1.278–2.926; *P* < .001, HR = 1.816, 95% CI: 1.470–2.243; *P* < .001, HR = 1.805, 95% CI: 1.380–2.362; *P* < .001, HR = 2.924, 95% CI: 2.189–3.905, Fig. 4C). And the multivariate Cox regression analysis demonstrated that age, N status and risk score based on the TRP-related lncRNAs signature were independent predictor of OS for BC patients (*P* = .021, HR = 1.643, 95% CI: 1.078–2.504; *P* = .026, HR = 1.406, 95% CI: 1.042–1.898; *P* < .001, HR = 2.561, 95% CI: 1.915–3.423, Fig. 4F).

### 3.4. Establishment of the nomogram in breast cancer

**Figure 5.**
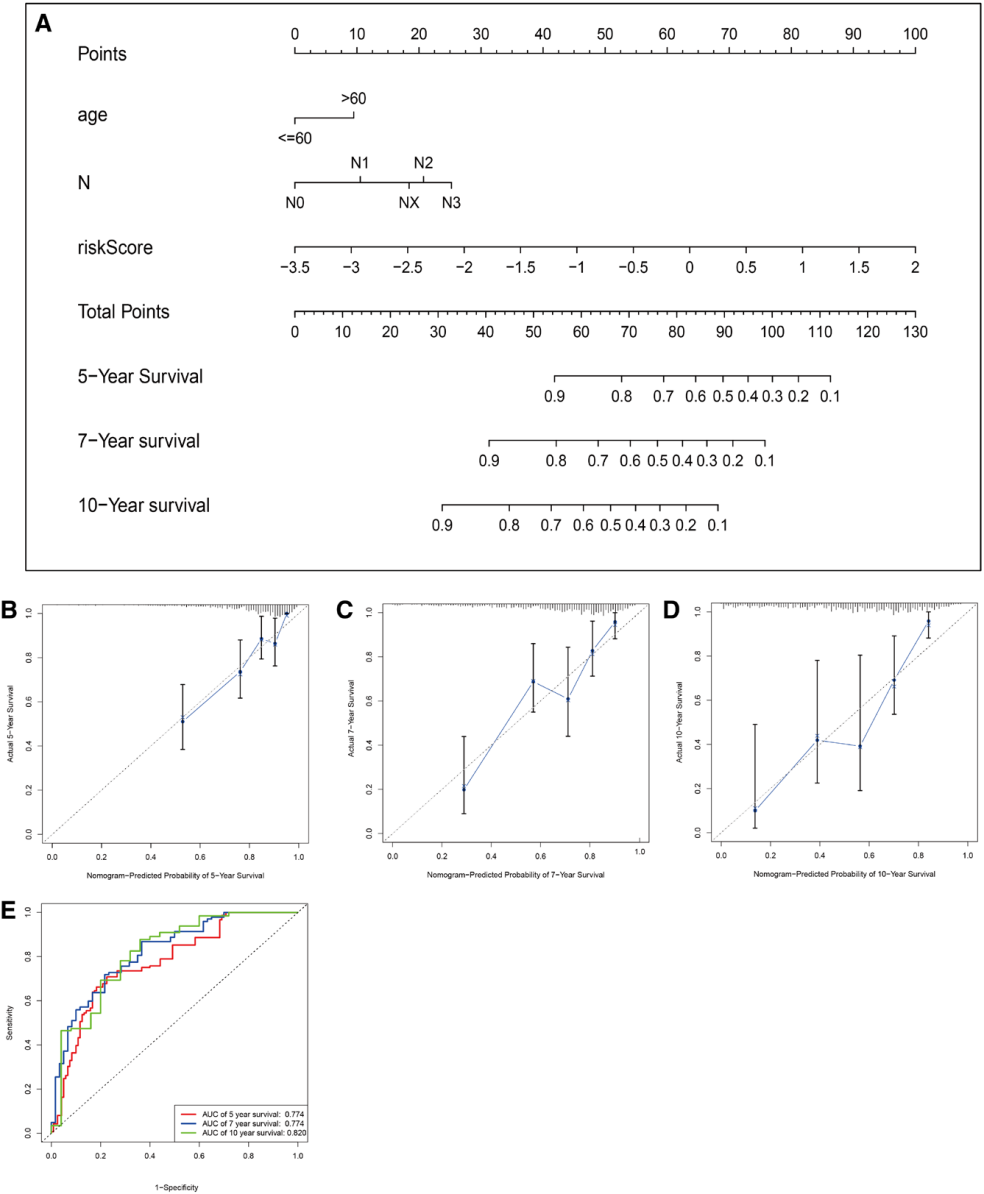
Clinical prognostic nomogram for survival prediction. (A) Clinical prognostic nomogram was applied to predict 5-, 7-, and 10-yr survival by age, lymph node status and risk score. Calibration curves showing nomogram predictions for 5-yr (B), 7-yr (C), and 10-yr (D) survival. (E) Time-dependent ROC curve analysis for predicting OS at 5-, 7-, and 10-year survival. OS = overall survival, ROC = receiver operating characteristic.

The nomogram was constructed to predict the 5-, 7-, and 10-years OS of BC patients based on the risk score and other independent prognostic factors in the TCGA data (Fig. 5A). The calibration curve revealed good performance of the nomogram for forecasting the 5-, 7-, and 10-years OS in breast cancer patient samples (Fig. 5B, C, and D). The AUC of the nomogram was 0.774, 0.774 and 0.820 for the 5-, 7- and 10-year OS, respectively (Fig. 5E).

### 3.5. The lncRNA–mRNA co-expression network and functional enrichment analysis

**Figure 6.**
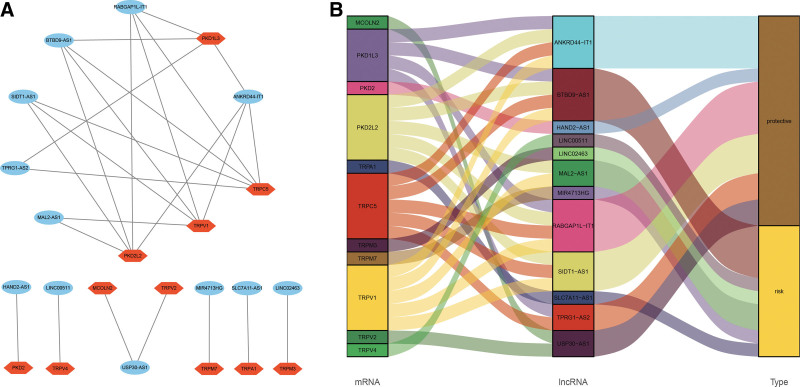
Establishment of a LncRNA-mRNA co-expression network (A). The relationship among 12 TRP-associated lncRNAs, 11 mRNAs, and risk types (risk or protective) were shown in Sankey diagram (B). lncRNA = long noncoding RNA, TRP = transient receptor potential.

A lncRNA-mRNA co-expression network contained 26 lncRNA-mRNA pairs was constructed to identify the potential prognostic roles of the 12 TRP-associated lncRNAs (Fig. 6A). The Sankey diagram not only displayed the relationship between 12 TRP-associated lncRNAs and targeted mRNAs, but also displayed the correlation between TRP-associated lncRNAs and the risk types (Fig. 6B).

**Figure 7.**
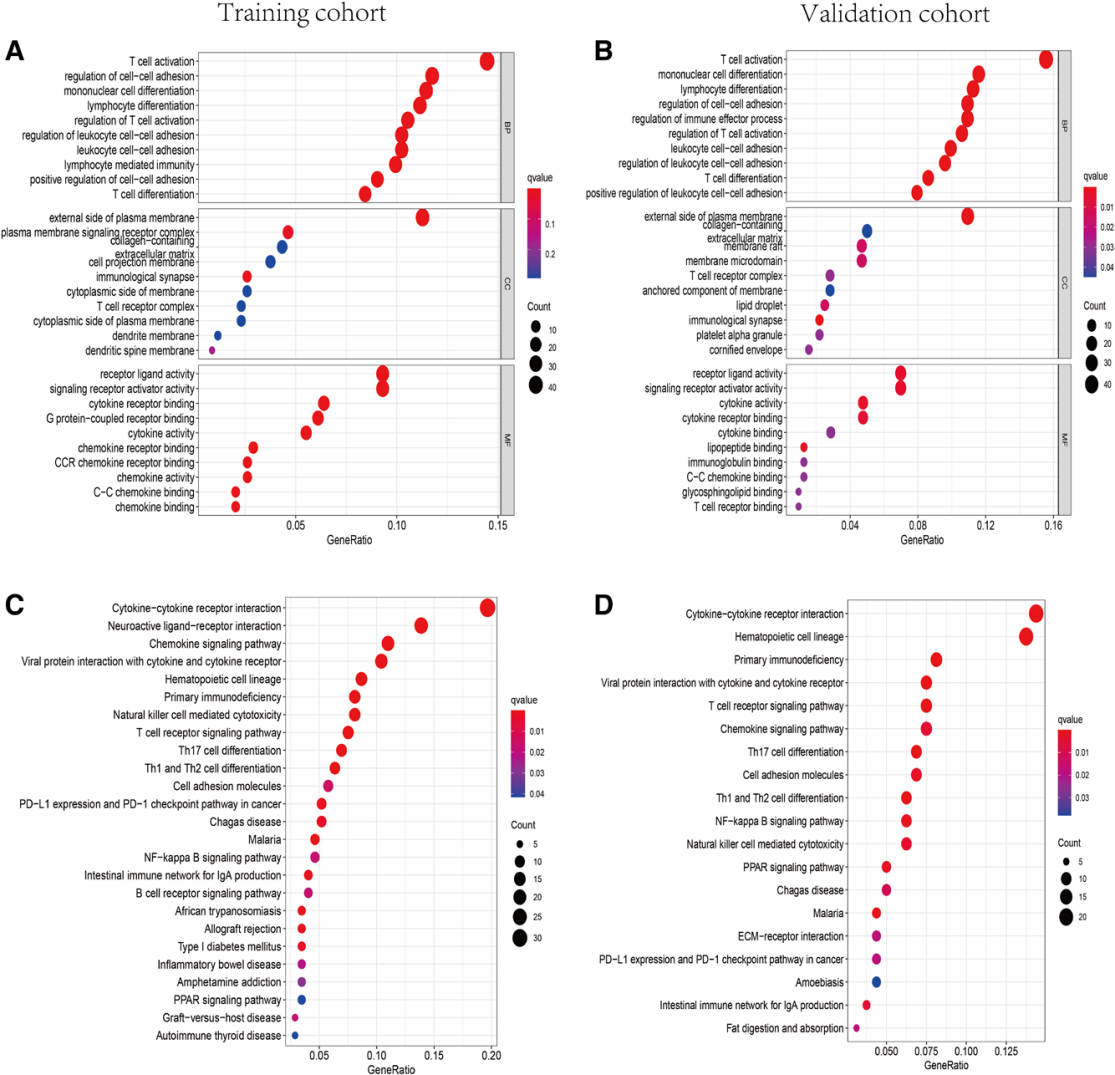
Representative results of GO and KEGG enrichment analysis in training and validation sets. (A, C) In training set, the DEGs between high-risk and low-risk groups enrichment in GO terms and KEGG pathways. (B, D) In validation set, the DEGs between different risk groups enrichment in GO terms and KEGG pathways. DEGs = differential expression genes, KEGG = Kyoto encyclopedia of genes and genomes.

GO and KEGG analysis were performed in the DEGs between high-risk and low-risk groups to investigate the biological processes and signaling pathways related to the TRP-associated-lncRNA signature. The top 30 GO terms were presented in Figure 7A and B. Besides, 25 and 19 KEGG enriched signing pathways in training and validation groups were presented in Figure 7C and D. Consequently, the results of GO and KEGG enrichment analysis were associated with immune cell activation, immunoregulation, and immune response.

### 3.6. Relevance of ESTIMATE scores and 12-TRP-related-lncRNA signature

**Figure 8.**
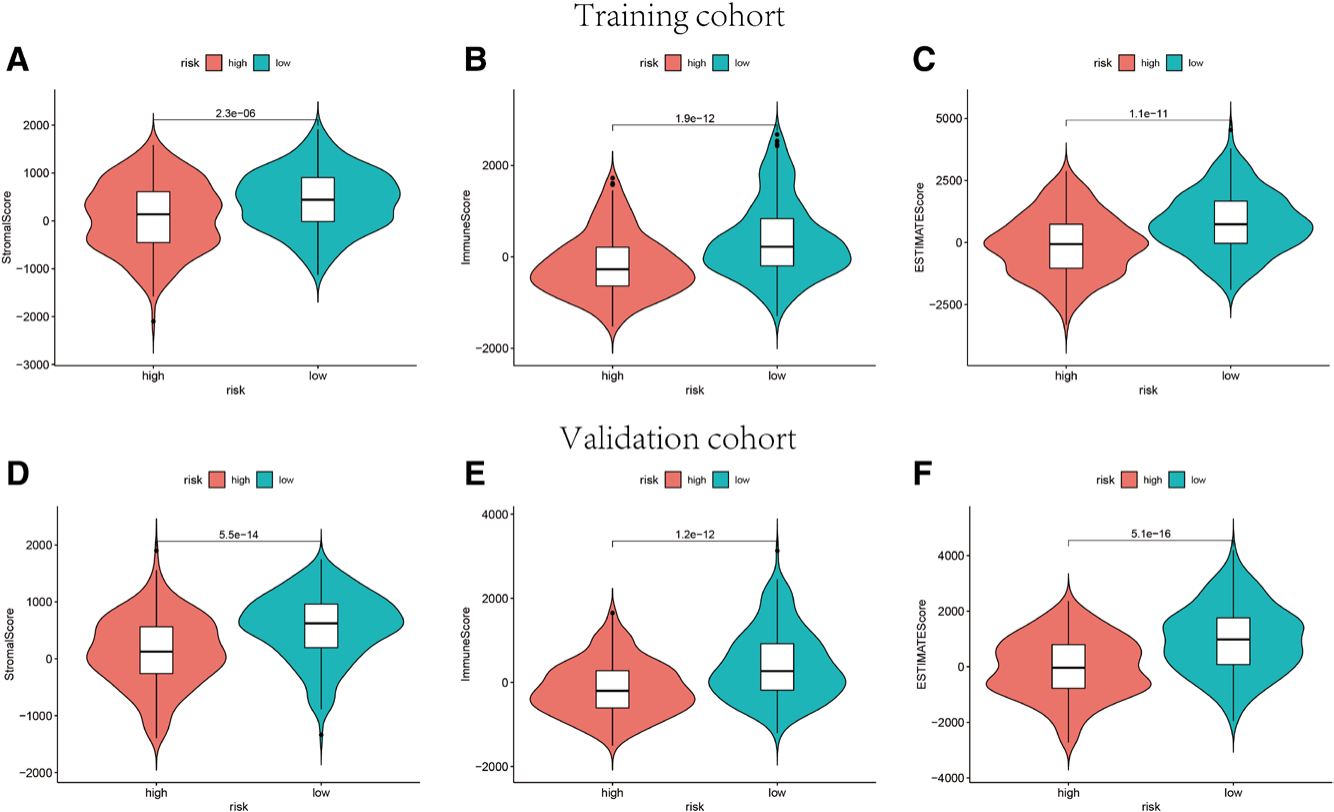
The ESTIMATE analysis. he stromal scores, immune scores, and ESTIMATE scores were significantly different between high-risk group and low-risk group in training (A, B and C) and validation (D, E and F) cohorts. ESTIMATE = Estimation of Stromal and Immune cells in Malignant Tumor tissues using expression.

The Stromal score, Immune score and ESTIMATE score for each sample were calculated by use of ESTIMATE algorithm to further descript the TIME landscape and the overall degree of immune infiltration. As a result, samples of low-risk group were indicated with higher Stromal score, Immune score, and ESTIMATE score instead of high-risk group samples in training set as well as validation set (*P* < .05) (Fig. 8).

### 3.7. Features of the tumor immune microenvironment in breast cancer

**Figure 9.**
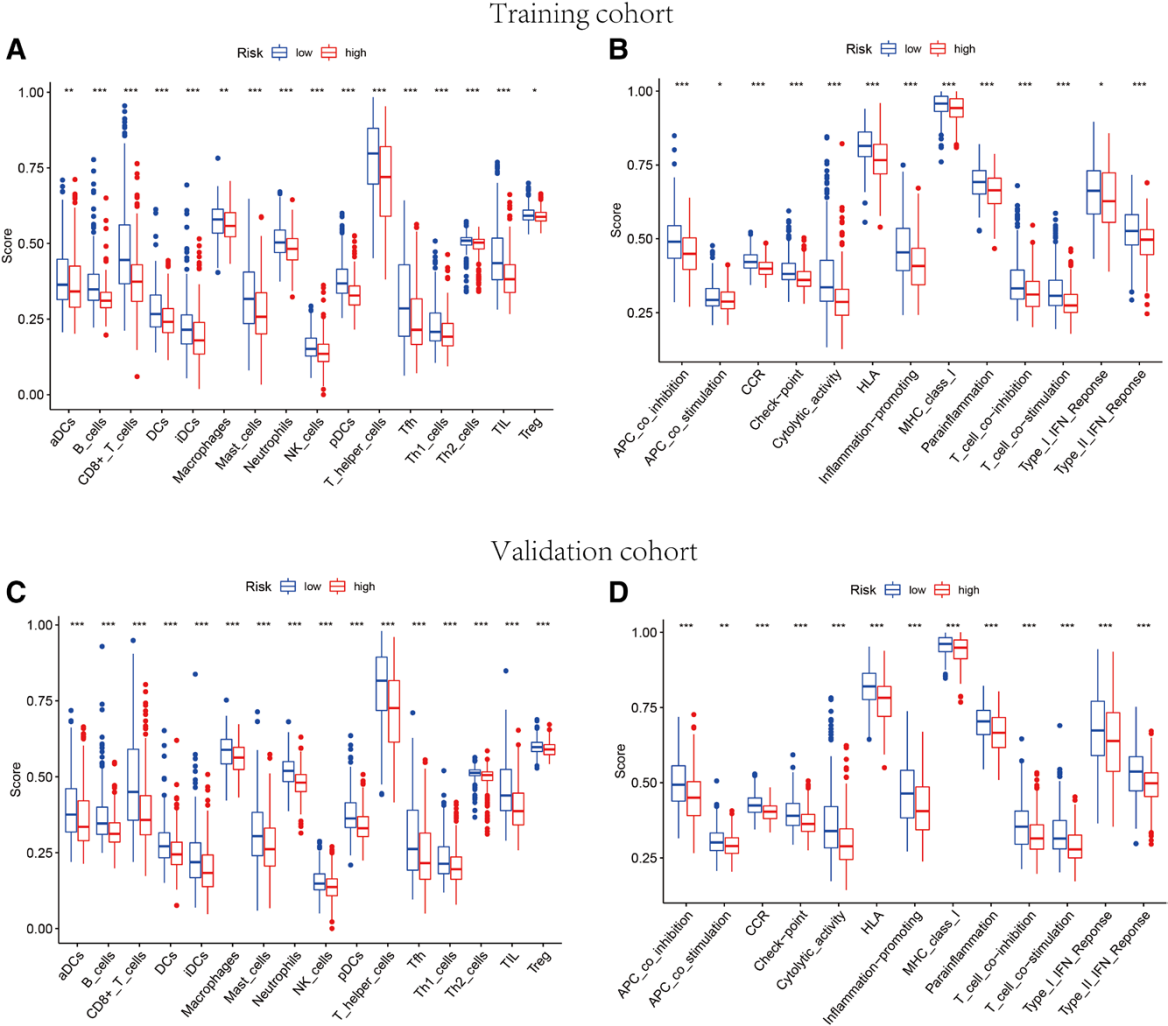
Relevance among TRP-associated-lncRNA risk score and TIME characteristics in BC. (A, C) The immune cell infiltration differences between high-risk group and low-risk group in training (A) and validation (C) cohorts. (B, D) The distinction of immune-associated signature between different risk groups in training (C) and validation (D) cohorts. (* indicated *P* < .05, ** indicated *P* < .01, *** indicated *P* < .001). BC = breast cancer, lncRNA = long noncoding RNA, TIME = tumor immune microenvironment, TRP = transient receptor potential.

According to the functional enrichment analysis, the DEGs between high-risk and low-risk groups were commonly enriched in some immune activity pathways. Hence, ssGSEA was employed to further investigate the differences of immune signatures for different risk groups. Both outcomes in training set and validation set revealed that the infiltration levels of some sorts of immune cell subtypes were notably increased as the risk scores decreased both in training and validation sets, for instance B cells, CD8 + T cells, DCs, invasive ductal carcinomas, Mast cells, Neutrophils, NK cells, pDCs, T helper cells, Tfh, Th1 cells, Th2 cells, TIL, aDCs and Macrophages (Fig. 9A and C). Additionally, in training and validation sets, several immune signatures were remarkably different between high-risk and low-risk groups, including APC co-inhibition, CCR, checkpoint, cytolytic activity, HLA, inflammation promoting, MHC class I, para-inflammation, T cell co-inhibition, T cell co-stimulation and IFN response type II (Fig. 9B and D).

### 3.8. Immunotherapy response prediction

**Figure 10.**
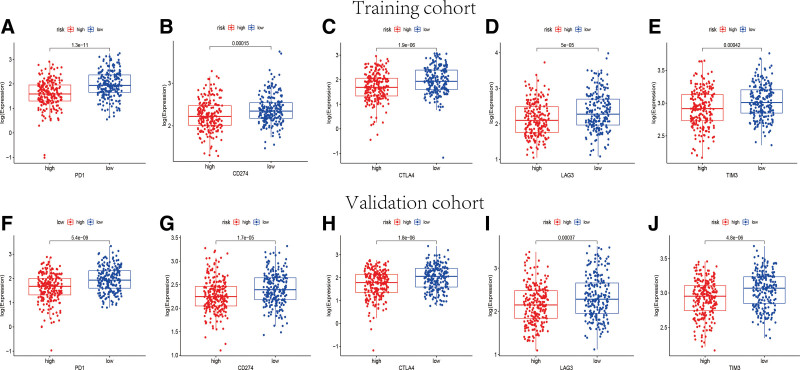
The different expression levels of immune checkpoint genes between high-risk and low- risk groups. The expression levels of PD1 (A), CD274 (B), CTLA4 (C), LAG3 (D) and TIM3 (E) in high- and low- risk groups in training set (*P* < .01). The expression levels of PD1 (F), CD274 (G), CTLA4 (H), LAG3 (I) and TIM3 (J) in high- and low-risk groups in validation set (*P* < .01).

**Figure 11.**
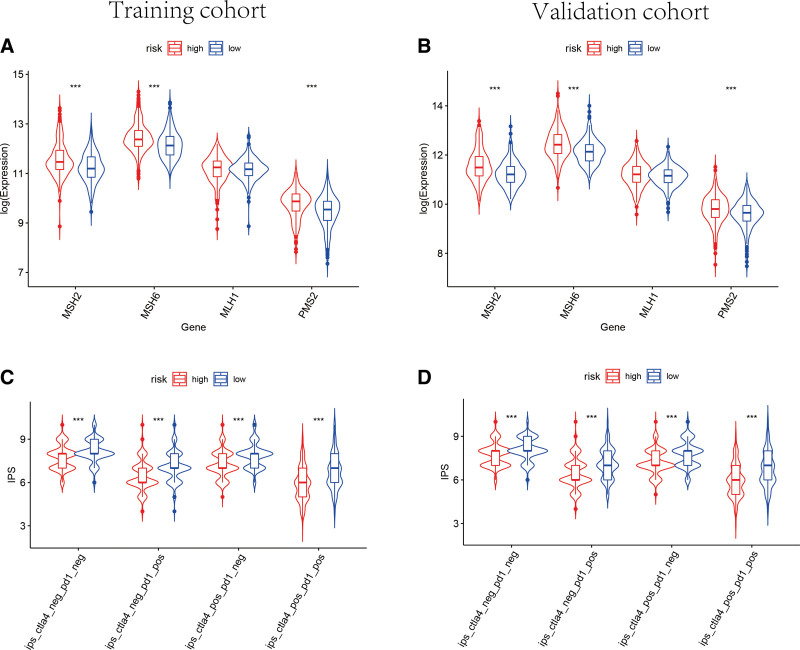
Immunotherapy response prediction. The expression of mismatch repair genes in breast cancer samples, including MSH2, MSH6, MLH1, and PMS2, expressed significantly lower in the low-risk groups in training (A) and validation (B) sets. The IPS of anti-(CTLA-4 plus PD-1), anti-CTLA-4, and anti-PD-1 in low-risk groups were notably higher in training (C) and validation (D) sets, indicating that BC patients with lower risk score shown better outcomes of immunotherapy responses. BC = breast cancer, IPS = immunophenoscore.

ICB molecules and MSI in tumor tissue were considered as potential biomarkers for immunotherapy response predicting. As shown in Figure 10, the expression levels of 5 key ICB genes were elevated in low-risk group both in training set and validation set, including PD1, CD274, LAG3, CTLA4 as well as TIM3(*P* < .05). simultaneously, the transcriptional expression of important mismatch repair genes in each tumor specimen were assessed and compared. MSH2, MSH6, and PMS2 were found highly expressed in the high-risk group instead of low-risk group (*P* < .001) (Fig. 11A and B). Accordingly, the microsatellites were considered more stable in the samples of high-risk group. IPS was a significant predictor for response to anti-PD-1 and anti-CTLA4. Therefore, the IPS of each sample was generated by use of The Cancer Immunome Atlas database to forecast the differences of immunotherapy response between high- and low- risk groups. The IPS of anti-PD-1, anti i-CTLA-4, and anti-CTLA-4 plus anti-PD-1 in low-risk group were significantly higher than that in high-risk group (Fig. 11C and D). Subsequently, the high-risk groups were observed greatly higher tumor mutation burden in both training cohort and validation cohort (Supplementary Fig. 4, http://links.lww.com/MD/K648). Consequently, the 12-TRP-related-lncRNA risk model had a potential ability to predict the immunotherapy response of BC patients. The patients with lower risk score were considered to have better outcomes with immunotherapy.

## 4. Discussion

Most of the multifunctional family of ion channels formed by TRP channels were calcium-permeable, shown intricate patterns of regulation and were sensitive to a wide range of environmental conditions. Some studies indicated that ion channels may hold a key role in the induction and progression of carcinoma.^[32]^ Several studies in breast cancer revealed that potassium (K+) channels^[33,34]^ were engaged in the processes of cell proliferation, cell cycle progression, as well as metastasis. Fraser et al found that the activities of sodium (Na+) channels^[35]^ were associated with the progression and invasion of breast carcinoma. Since 1969, TRP channels were firstly discovered in drosophila by Cosens and Manning,^[36]^ the role of TRP channels in malignant tumors continues to be identified. The roles of different TRP subfamily members were varied in different kinds of neoplasm. Gkika and colleagues conducted a study of TRP channels in prostate cancer,^[37]^ suggesting that TRPM8 and TRPV6 were novel markers for tumor progression. In hepatocellular carcinoma, TRPV2 was identified as a potential prognostic factor due to the correlation of tumor differentiation and TPRV2 gene expression.^[38]^ He B et al found that the invasion abilities of nasopharyngeal cancer cells could be inhibited by silencing the expression of TRPC1 in tumor cells.^[17]^ Besides, the pharmacological inhibitors for TPRC1 could make the cell cycle of malignant glioma stagnating to suppress the tumor cell proliferation.^[18]^ In esophageal carcinoma, TRPC6 was proposed as a potential target for treatment by Ding and colleagues, which was associated with the growth of tumor cells.^[39]^

Breast cancer is a kind of highly heterogeneous tumor with a high rate of proliferation, invasion, and metastasis, which was associated with the microenvironment changes.^[40]^ The development of mammary tumor was considered associated with the deregulation of Ca2 + homeostasis.^[41,42]^ In 2003, the calcium channels were firstly discovered to play a regulator role in breast tumor malignant transformation.^[42]^ After that, the correlation between Ca2 + homeostasis and breast tumor progression were further explored. Some TRP subfamily members were suggested to play vital roles in growth and migration of BC cells, including TRPM7,^[43]^ TRPC1,^[44]^ and TPRV6.^[45]^ In addition, a number of academics investigated in the relationship among TRP channels and clinical characteristics in BC. Recently, several TRPs were reported highly overexpressed in invasive ductal carcinoma, for example TRPC6, TRPM7, TRPM8, TRPV6 and TRPC1.^[46,47]^ As such, TRPs were proposed by some scholars to be play as novel biomarkers for BC diagnosis and treatment. Despite of multiple approaches were developed for antitumor, patients suffered from advanced BC were still considered a big medical challenge. Recently, more and more academics focused on the potential ability of TRP channel to be as the targets of anticancer therapy. However, knowledge about the relationship between TPR channels and immunotherapy is lacking. Hence, we explored the correlation of TRP-related lncRNAs and the prognostic outcomes of BC patients to identify the potential targets for BC immunotherapy.

In our study, the prognostic lncRNAs associated with TRP channels were identified by the analysis of lncRNA expression in BC samples from TCGA data. After that, the 12-TRP-related-lncRNA risk model was established to divide the BC patients in training set and validation set into high-risk groups and low-risk groups according to the median cutoff value of training set. Interestingly, a significant distinction in prognostic outcomes was observed between different risk groups via survival analysis. The AUCs of the 12-lncRNA-based risk scores in training ser for the 5-, 7- and 10-year OS predictors were 0.752, 0.822 and 0.833, respectively. Subsequently, we created a lncRNA-mRNA co-expression network and conducted functional enrichment analysis. Notably, biological processes associated with immunology was identified highly enriched, including but not limited to immune cell activation and immune response. Therefore, the infiltration of immune cells was further investigated to identify the features of TRP-related tumor immune microenvironment.

Recently, some academics focused on the role of TRP channels in calcium signaling and immunomodulation. Calcium is core to a number of biological processes, for example, the activation and maintenance of the immune system.^[26]^ As of now, store-operated calcium entry (SOCE) was the most widely known mechanism of calcium ion infusion into cells, that was the key to immune cell activation.^[48]^ TRP channels were engaged in a variety of biological processes in the immune system, involving activation of B and T cell receptor, antigen presentation via DCs, degranulation of mast cell as well as bactericidal activities of macrophage and neutrophil.^[26]^ Some studies indicated that TRP channels were significant for the initiation of adaptive and innate immune response. Several studies proved that the TRP channels were expressed both in the murine T cells^[49,50]^ and human T cells,^[51]^ such as TRPV, TRPC, TRPA and TRPM. TRPC5 was identified as an immunosuppressor associated with CD4 + and CD8 + T cell activation.^[52]^ TPRC3 was reported to be upregulated in response to the stimulation of multiclonal T cells, further promoting the proliferation of cells associated with Ca2+.^[51]^ Moreover, some studies indicated that TRPV1 and TRPV2 were expressed not only in human leukocytes/lymphocytes but also in CD4 + T cells.^[53,54]^ Besides, several TRP channels were suggested to be expressed in primary human B cells, including TRPC2, TRPC6, TPRV2 and TPRM7.^[55–57]^ TRPC channels were demonstrated to be involved in B cell receptor signaling.^[55]^ Simultaneously, TPRM channels were found to play important roles in cell proliferation and intracellular tracking regulation. As for innate immune system, recent research revealed that TRP channels were involved in the Ca2 + homeostasis in NK cells.^[28,58]^ TRPV and TRPM channels were identified linked to DCs function, including thermosensation, antigen presentation, trafficking and migration.^[26,59]^ In addition, there was increasing evidence that TRP channels were essential for monocytes, macrophages, neutrophils, and mast cells.^[26]^ TRPC1 has been demonstrated associated with neutrophil migration in murine.^[60]^ TRPV2 was reported to be a participant in macrophage chemotaxis and cytokine production.^[61,62]^ The study in asthmatic rats revealed that TRPM7 channel played a key in mast cell function, for instance, degranulation and cytokine release.^[63]^ According to our research, TRP-related lncRNA risk model was associated with the infiltration of immune cells. Hence, we hypothesized that the expression of TRP channels could be closely related to alterations in the tumor immune microenvironment. However, seldom studies investigated the regulating mechanisms of TRP channels in TIME.

Since the outcomes linked the TRP-related lncRNA signature to immune cell infiltration in breast cancer, these TRP-related lncRNAs may be the novel targets for immunotherapies, such as immune checkpoint inhibitors. According to the results of our study, low-risk group based on 12-TRP-lncRNA risk model presented higher immune checkpoint molecules expression levels with better prognosis. Besides, IPS and MSI analysis were further indicated that lower risk scores meant better immunotherapy response. As such, the TRP-associated-lncRNA risk model was considered as a predictor for immunotherapy response in patients with breast cancer, which was able to facilitate the development of novel treatment strategies.

Our study was the first one to construct a TRP-associated-lncRNA risk model based on 12 TRP-related lncRNAs by use of public databases, considered as independent factor for prognosis predicter in patients with breast cancer. However, there are still some limitations in our study. Firstly, a single data collected from TCGA database was enrolled in this study. Secondly, due to the incomplete clinical information, some other prognostic features were excluded out univariate and multivariate COX regression analysis, for example immunotherapy, chemotherapy, and radiotherapy data. Thirdly, this study only explored the relationship between the TRP-associated-lncRNA signature and infiltration of immune cells, while seldom investigated the immune regulating mechanisms of TRP channels. Fourthly, the training set and validation set were randomly divided, so the constructed signature might vary from different groupings. Although the predictive efficiency of the signature was also validated in the whole dataset to reduce the bias caused by random grouping, the better constructed prognostic signatures are still possible. We do not deny the randomness of the model. Consequently, additional validation experiments are essential to confirm the prognostic predictive efficiency of this signature and the correlation of TRP channels and tumor immune microenvironment should be further studied. We provided a novel insight for anti-tumor immunity in breast cancer.

## 5. Conclusion

A TRP-related lncRNAs risk model was constructed as an independent prognostic risk factor for breast cancer patients by use of bioinformatics tools and associated algorithms. This model is believed to be closely related to the immune cell infiltration and plays a vital role in new strategies for the future immunotherapy of breast cancer.

## Acknowledgements

The authors would like to thank TCGA (http://cancergenome.nih.gov) for data collection. A preprint has previously been published [Qiaonan Guo, Pengjun Qiu, Kelun Pan, Jianpeng Chen, Baiwei Wang and Jianqing Lin. 2022]^[64]^

## Author contributions

**Conceptualization:** Qiaonan Guo, Pengjun Qiu, Jianqing Lin.

**Data curation:** Kelun Pan, Jianpeng Chen, Baiwei Wang.

**Formal analysis:** Pengjun Qiu.

**Methodology:** Qiaonan Guo, Pengjun Qiu, Kelun Pan.

**Software:** Pengjun Qiu.

**Writing – original draft:** Qiaonan Guo.

**Writing – review & editing:** Pengjun Qiu, Jianqing Lin.

## Supplementary Material














