# Supplementary material for: Construction and validation of a transient receptor potential-related long noncoding RNA signature for prognosis prediction in breast cancer patients
Source: Medicine (Baltimore). 2023 Nov 17;102(46):e35978. doi: 10.1097/MD.0000000000035978 (PMC10659707; doi:10.1097/MD.0000000000035978)
Supplement: Supplementary file 1 [file medi-102-e35978-s001.pdf]

Table S1. TRP-related Genes

TRPC1
TRPC2
TRPC3
TRPC4
TRPC5
TRPC6
TRPC7
TRPV1
TRPV2
TRPV3
TRPV4
TRPV5
TRPV6
TRPM1
TRPM2
TRPM3
TRPM4
TRPM5
TRPM6
TRPM7
TRPM8
TRPA1
MCOLN1
MCOLN2
MCOLN3
PKD1
PKD2
PKD2L1
PKDREJ
PKD2L2
PKD1L1
PKD1L2
PKD1L3
