# Supplementary material for: Construction and validation of a transient receptor potential-related long noncoding RNA signature for prognosis prediction in breast cancer patients
Source: Medicine (Baltimore). 2023 Nov 17;102(46):e35978. doi: 10.1097/MD.0000000000035978 (PMC10659707; doi:10.1097/MD.0000000000035978)
Supplement: Supplementary file 2 [file medi-102-e35978-s002.pdf]

Table S2. The TRP-related lncRNAs identified by Pearson analysis

TRP	lncRNA	cor	pvalue
TRPC5	DHDDS-AS1	0.4933546	8.37E-55
TRPV1	DHDDS-AS1	0.5418239	7.76E-68
PKD2L2	DHDDS-AS1	0.5322014	4.44E-65
PKD1L3	DHDDS-AS1	0.4850084	9.18E-53
TRPV2	PIK3CD-AS1	0.4826176	3.44E-52
MCOLN2	PIK3CD-AS1	0.5297602	2.15E-64
TRPC5	RERE-AS1	0.6725842	3.84E-116
TRPV1	RERE-AS1	0.5867152	5.81E-82
PKD2L2	RERE-AS1	0.6177739	4.17E-93
PKD1L3	RERE-AS1	0.6676323	7.38E-114
PKD2L2	ZMYM4-AS1	0.5428657	3.85E-68
TRPC5	C1orf195	0.6142029	9.22E-92
TRPV1	C1orf195	0.697976	1.37E-128
PKD2L2	C1orf195	0.5793883	1.66E-79
PKD1L3	C1orf195	0.6896218	2.36E-124
TRPM1	MIR3659HG	0.671607	1.09E-115
PKD1	LINC01786	0.4432694	2.29E-43
TRPC5	LINC01398	0.565621	4.70E-75
TRPV1	LINC01398	0.4249411	1.23E-39
PKD1L3	LINC01398	0.5010737	9.67E-57
TRPV2	LINC02812	0.4348862	1.24E-41
MCOLN2	LINC02812	0.4987038	3.85E-56
TRPC5	TXNDC12-AS	0.5530328	3.65E-71
TRPV1	TXNDC12-AS	0.5341495	1.25E-65
PKD2L2	TXNDC12-AS	0.5165824	8.69E-61
PKD1L3	TXNDC12-AS	0.5061716	4.77E-58
TRPC5	NFIA-AS1	0.4288587	2.05E-40
TRPV1	NFIA-AS1	0.4230819	2.86E-39
PKD2L2	NFIA-AS1	0.7044506	5.61E-132
PKD2L2	DLEU2L	0.4265982	5.79E-40
TRPC5	ERI3-IT1	0.6354838	4.85E-100
TRPV1	ERI3-IT1	0.6890246	4.68E-124
PKD2L2	ERI3-IT1	0.5668151	1.97E-75
PKD1L3	ERI3-IT1	0.599555	2.02E-86
MCOLN2	LINC01781	0.4171103	4.14E-38
TRPC5	LINC01359	0.5246975	5.46E-63
TRPV1	LINC01359	0.5815437	3.20E-80
PKD2L2	LINC01359	0.6312239	2.48E-98
PKD1L3	LINC01359	0.4577494	1.76E-46
PKD2L2	PDE4B-AS1	0.4757865	1.42E-50
TRPV3	PRKACB-DT	0.4211796	6.74E-39
TRPC5	TTLL7-IT1	0.4160424	6.63E-38
TRPM3	LINC01748	0.4252874	1.05E-39
PKD2	GNG12-AS1	0.4066235	3.96E-36
TRPC5	FAF1-AS1	0.6896416	2.31E-124
TRPV1	FAF1-AS1	0.5882451	1.75E-82
PKD2L2	FAF1-AS1	0.6227744	5.09E-95
PKD1L3	FAF1-AS1	0.5866724	6.01E-82
MCOLN2	LINC01358	0.4116607	4.52E-37

TRPC5	DPYD-AS1	0.5794473	1.59E-79
TRPV1	DPYD-AS1	0.496709	1.22E-55
PKD2L2	DPYD-AS1	0.5995104	2.09E-86
PKD1L3	DPYD-AS1	0.5492256	5.09E-70
MCOLN2	SLC16A1-AS	0.4502893	7.39E-45
TRPC5	KIRREL1-IT	0.6308082	3.63E-98
TRPV1	KIRREL1-IT	0.6618364	3.06E-111
PKD2L2	KIRREL1-IT	0.5028108	3.49E-57
PKD1L3	KIRREL1-IT	0.6379213	4.97E-101
TRPC5	PBX1-AS1	0.6366692	1.61E-100
TRPV1	PBX1-AS1	0.475917	1.32E-50
PKD2L2	PBX1-AS1	0.5867437	5.69E-82
PKD1L3	PBX1-AS1	0.5682134	7.08E-76
TRPC5	KIF26B-AS1	0.4130674	2.45E-37
TRPV1	KIF26B-AS1	0.507031	2.86E-58
PKD2L2	KIF26B-AS1	0.4266896	5.56E-40
PKD1L3	KIF26B-AS1	0.5279705	6.80E-64
TRPM8	LINC02608	0.5325808	3.47E-65
TRPC5	SMYD3-IT1	0.5411338	1.23E-67
TRPV1	SMYD3-IT1	0.4822833	4.14E-52
PKD2L2	SMYD3-IT1	0.5845554	3.13E-81
PKD1L3	SMYD3-IT1	0.4546265	8.52E-46
TRPC5	RGS5-AS1	0.4568028	2.85E-46
TRPV1	RGS5-AS1	0.4815567	6.17E-52
PKD2L2	RGS5-AS1	0.420123	1.08E-38
PKD1L3	RGS5-AS1	0.4785558	3.18E-51
TRPV4	LEMD1-DT	0.4098635	9.84E-37
TRPC5	SMYD3-AS1	0.4440601	1.56E-43
TRPC5	LYST-AS1	0.6164672	1.30E-92
TRPV1	LYST-AS1	0.5557127	5.60E-72
PKD2L2	LYST-AS1	0.5618496	7.17E-74
PKD1L3	LYST-AS1	0.5583051	8.98E-73
TRPC5	RABGAP1L-A	0.4625139	1.54E-47
TRPV1	RABGAP1L-A	0.4434564	2.09E-43
PKD2L2	RABGAP1L-A	0.5827321	1.28E-80
PKD1L3	RABGAP1L-A	0.4544323	9.39E-46
TRPC5	RABGAP1L-I	0.445938	6.27E-44
TRPV1	RABGAP1L-I	0.4164768	5.47E-38
PKD2L2	RABGAP1L-I	0.5953749	6.01E-85
PKD1L3	RABGAP1L-I	0.4471879	3.40E-44
TRPV4	SERTAD4-AS	0.4073356	2.92E-36
TRPC5	MIR181A1HG	0.487068	2.92E-53
TRPV1	MIR181A1HG	0.4585309	1.18E-46
PKD2L2	MIR181A1HG	0.5052462	8.26E-58
PKD1L3	MIR181A1HG	0.4555213	5.43E-46
MCOLN2	MDS2	0.4349264	1.22E-41
TRPC5	MIR29B2CHG	0.5196466	1.30E-61
TRPV1	MIR29B2CHG	0.4973228	8.57E-56
PKD2L2	MIR29B2CHG	0.4150662	1.02E-37
PKD1L3	MIR29B2CHG	0.4267824	5.33E-40
TRPC5	ASH1L-IT1	0.6663873	2.72E-113

TRPV1	ASH1L-IT1	0.5919624	9.25E-84
PKD2L2	ASH1L-IT1	0.6870127	4.65E-123
PKD1L3	ASH1L-IT1	0.6185484	2.12E-93
TRPV1	MKNK1-AS1	0.4725877	7.88E-50
TRPV1	PABPC4-AS1	0.4594568	7.39E-47
PKD2L2	PABPC4-AS1	0.4043047	1.06E-35
TRPC5	ADAMTSL4-A	0.7157486	4.10E-138
TRPV1	ADAMTSL4-A	0.7553276	3.28E-162
PKD2L2	ADAMTSL4-A	0.5416855	8.51E-68
PKD1L3	ADAMTSL4-A	0.7504175	5.64E-159
TRPC5	LINC02809	0.6578929	1.71E-109
TRPV1	LINC02809	0.7140042	3.80E-137
PKD2L2	LINC02809	0.5981745	6.23E-86
PKD1L3	LINC02809	0.7684105	3.23E-171
PKD2	LINC01352	0.4287687	2.14E-40
PKD2	HLX-AS1	0.4134872	2.04E-37
TRPC5	RC3H1-IT1	0.7966812	6.99E-193
TRPV1	RC3H1-IT1	0.6920477	1.44E-125
PKD2L2	RC3H1-IT1	0.5741917	8.43E-78
PKD1L3	RC3H1-IT1	0.7471142	7.67E-157
TRPC5	KCND3-AS1	0.8432385	2.65E-237
TRPV1	KCND3-AS1	0.661714	3.47E-111
PKD2L2	KCND3-AS1	0.545322	7.33E-69
PKD1L3	KCND3-AS1	0.6496986	6.07E-106
TRPC5	ITPKB-IT1	0.4249775	1.21E-39
PKD1L3	ITPKB-IT1	0.476374	1.03E-50
TRPC5	ITPKB-AS1	0.7391767	7.57E-152
TRPV1	ITPKB-AS1	0.6613422	5.09E-111
PKD2L2	ITPKB-AS1	0.6038505	5.86E-88
PKD1L3	ITPKB-AS1	0.7516048	9.46E-160
TRPC5	NECTIN4-AS	0.4987604	3.73E-56
TRPV1	NECTIN4-AS	0.609999	3.36E-90
PKD1L3	NECTIN4-AS	0.6058133	1.14E-88
TRPC5	SNAP47-AS1	0.631932	1.30E-98
TRPV1	SNAP47-AS1	0.5526823	4.66E-71
PKD2L2	SNAP47-AS1	0.4317579	5.37E-41
PKD1L3	SNAP47-AS1	0.5913507	1.51E-83
TRPC5	EFCAB14-AS	0.4018772	2.96E-35
TRPV1	EFCAB14-AS	0.4237055	2.16E-39
PKD2L2	EFCAB14-AS	0.4095413	1.13E-36
TRPC5	AKT3-IT1	0.4034966	1.49E-35
TRPV1	AKT3-IT1	0.4906778	3.83E-54
PKD2L2	AKT3-IT1	0.5385942	6.69E-67
PKD1L3	AKT3-IT1	0.5153648	1.84E-60
PKD1L3	LINC01409	0.411444	4.96E-37
PKD1L3	ECE1-AS1	0.4055535	6.25E-36
TRPC5	MTOR-AS1	0.6979136	1.47E-128
TRPV1	MTOR-AS1	0.75347	5.61E-161
PKD2L2	MTOR-AS1	0.6790104	3.57E-119
PKD1L3	MTOR-AS1	0.6728709	2.82E-116
TRPV2	TFAP2E-AS1	0.5448918	9.81E-69

MCOLN2	TFAP2E-AS1	0.4994733	2.46E-56
TRPV1	ZRANB2-AS1	0.4057588	5.72E-36
TRPM3	LINC02765	0.4528745	2.05E-45
TRPC5	CDC42-IT1	0.6703453	4.19E-115
TRPV1	CDC42-IT1	0.6798538	1.41E-119
PKD2L2	CDC42-IT1	0.6622233	2.06E-111
PKD1L3	CDC42-IT1	0.631654	1.67E-98
MCOLN2	LINC01871	0.509833	5.32E-59
TRPC5	LINC00954	0.4387079	2.04E-42
PKD1L3	LINC00954	0.4340783	1.82E-41
TRPC5	MAPRE3-AS1	0.8892741	1.74E-298
TRPV1	MAPRE3-AS1	0.7392774	6.56E-152
PKD2L2	MAPRE3-AS1	0.5870947	4.32E-82
PKD1L3	MAPRE3-AS1	0.8314147	9.55E-225
TRPV1	MEIS1-AS2	0.4309888	7.67E-41
PKD2L2	MEIS1-AS2	0.4227729	3.29E-39
PKD1L3	MEIS1-AS2	0.4264434	6.22E-40
TRPC5	PRKCE-AS1	0.9013617	3.56952547807384e-319
TRPV1	PRKCE-AS1	0.7243076	5.76E-143
PKD2L2	PRKCE-AS1	0.6248288	8.15E-96
PKD1L3	PRKCE-AS1	0.8014398	7.22E-197
TRPC5	SLC8A1-AS1	0.5142825	3.57E-60
PKD2L2	SLC8A1-AS1	0.4818107	5.37E-52
PKD1L3	SLC8A1-AS1	0.4668335	1.64E-48
TRPC5	EVA1A-AS	0.74016	1.86E-152
TRPV1	EVA1A-AS	0.6940858	1.34E-126
PKD2L2	EVA1A-AS	0.5392409	4.35E-67
PKD1L3	EVA1A-AS	0.771816	1.17E-173
TRPV2	EML4-AS1	0.4745347	2.78E-50
MCOLN2	EML4-AS1	0.5019864	5.66E-57
TRPM6	LINC01143	0.4058299	5.55E-36
MCOLN2	LINC01800	0.4263219	6.57E-40
TRPV1	TCF7L1-IT1	0.4157117	7.67E-38
PKD2L2	TCF7L1-IT1	0.4595425	7.08E-47
PKD1L3	TCF7L1-IT1	0.4134119	2.11E-37
TRPC5	NPAS2-AS1	0.6966334	6.70E-128
TRPV1	NPAS2-AS1	0.4719925	1.08E-49
PKD2L2	NPAS2-AS1	0.518896	2.08E-61
PKD1L3	NPAS2-AS1	0.6376925	6.16E-101
TRPV4	LINC01956	0.4133862	2.13E-37
TRPC5	NCKAP5-IT1	0.4512558	4.58E-45
PKD2L2	NCKAP5-IT1	0.5196067	1.34E-61
TRPV2	SOCAR	0.429158	1.79E-40
TRPC5	ANKRD44-IT	0.7043436	6.39E-132
TRPV1	ANKRD44-IT	0.5703713	1.45E-76
PKD2L2	ANKRD44-IT	0.6305862	4.45E-98
PKD1L3	ANKRD44-IT	0.672749	3.21E-116
TRPC5	LIMS1-AS1	0.451602	3.86E-45
TRPV1	LIMS1-AS1	0.4635779	8.91E-48
PKD2L2	LIMS1-AS1	0.4413181	5.86E-43
PKD1L3	LIMS1-AS1	0.4485718	1.73E-44

MCOLN2	LINC01891	0.4084697	1.79E-36
TRPC5	KANSL1L-AS	0.4892013	8.81E-54
TRPV1	KANSL1L-AS	0.4564152	3.46E-46
PKD2L2	KANSL1L-AS	0.4599497	5.75E-47
PKD1L3	KANSL1L-AS	0.4728768	6.75E-50
TRPV2	LINC01934	0.5582917	9.07E-73
MCOLN2	LINC01934	0.5937695	2.18E-84
TRPC5	NCKAP5-AS1	0.7519921	5.27E-160
TRPV1	NCKAP5-AS1	0.5932499	3.31E-84
PKD2L2	NCKAP5-AS1	0.5191045	1.83E-61
PKD1L3	NCKAP5-AS1	0.6612384	5.66E-111
TRPV2	LINC01943	0.593244	3.33E-84
MCOLN2	LINC01943	0.4880079	1.72E-53
TRPC5	KLF7-IT1	0.4558718	4.55E-46
TRPV1	KLF7-IT1	0.4265972	5.80E-40
PKD1L3	KLF7-IT1	0.4237131	2.15E-39
PKD2	LINC01614	0.4223284	4.02E-39
TRPV2	LINC01857	0.6146992	6.01E-92
MCOLN2	LINC01857	0.5620305	6.29E-74
TRPC5	RHOQ-AS1	0.5896091	5.98E-83
TRPV1	RHOQ-AS1	0.6211833	2.09E-94
PKD2L2	RHOQ-AS1	0.5600859	2.53E-73
PKD1L3	RHOQ-AS1	0.6451133	5.27E-104
TRPC5	TTC21B-AS1	0.5284672	4.94E-64
TRPV1	TTC21B-AS1	0.6061291	8.76E-89
PKD2L2	TTC21B-AS1	0.6206722	3.28E-94
PKD1L3	TTC21B-AS1	0.5401439	2.39E-67
TRPV2	ZEB2-AS1	0.4755722	1.59E-50
TRPC5	GTF3C2-AS1	0.4654382	3.40E-48
TRPV1	GTF3C2-AS1	0.5335901	1.80E-65
PKD2L2	GTF3C2-AS1	0.4526209	2.32E-45
PKD1L3	GTF3C2-AS1	0.4654852	3.31E-48
TRPC5	CFLAR-AS1	0.6924394	9.12E-126
TRPV1	CFLAR-AS1	0.6654875	6.98E-113
PKD2L2	CFLAR-AS1	0.6551933	2.60E-108
PKD1L3	CFLAR-AS1	0.7417665	1.86E-153
TRPC5	MSH2-OT1	0.4102023	8.50E-37
PKD1L3	MSH2-OT1	0.4471062	3.54E-44
TRPV2	LINC02084	0.5104711	3.62E-59
MCOLN2	LINC02084	0.5252553	3.84E-63
TRPV1	CNTN4-AS2	0.4383262	2.45E-42
PKD2L2	CNTN4-AS2	0.4045385	9.62E-36
TRPC5	THRB-IT1	0.5768727	1.12E-78
TRPV1	THRB-IT1	0.4962217	1.62E-55
PKD2L2	THRB-IT1	0.6434965	2.50E-103
PKD1L3	THRB-IT1	0.4966441	1.27E-55
TRPV1	SLC6A1-AS1	0.4466876	4.35E-44
TRPC5	OSBPL10-AS	0.6815898	2.06E-120
TRPV1	OSBPL10-AS	0.5811512	4.32E-80
PKD2L2	OSBPL10-AS	0.6835771	2.24E-121
PKD1L3	OSBPL10-AS	0.6194626	9.50E-94

TRPC5	RBMS3-AS2	0.7192271	4.60E-140
TRPV1	RBMS3-AS2	0.6698023	7.46E-115
PKD2L2	RBMS3-AS2	0.7212589	3.23E-141
PKD1L3	RBMS3-AS2	0.6642714	2.47E-112
TRPC5	C3orf35	0.4503411	7.21E-45
TRPV1	C3orf35	0.4276069	3.65E-40
TRPC5	PRICKLE2-A	0.6361402	2.63E-100
TRPV1	PRICKLE2-A	0.5631999	2.71E-74
PKD2L2	PRICKLE2-A	0.6758579	1.12E-117
PKD1L3	PRICKLE2-A	0.5518879	8.10E-71
TRPC5	CACNA2D3-A	0.8379879	1.33E-231
TRPV1	CACNA2D3-A	0.4788145	2.76E-51
PKD1L3	CACNA2D3-A	0.6951739	3.74E-127
TRPC5	FOXP1-IT1	0.5932182	3.40E-84
TRPV1	FOXP1-IT1	0.5504179	2.24E-70
PKD2L2	FOXP1-IT1	0.7527445	1.69E-160
PKD1L3	FOXP1-IT1	0.4934854	7.77E-55
TRPC5	FOXP1-AS1	0.6956714	2.08E-127
TRPV1	FOXP1-AS1	0.6505062	2.74E-106
PKD2L2	FOXP1-AS1	0.7074335	1.44E-133
PKD1L3	FOXP1-AS1	0.5943099	1.42E-84
TRPC5	DENND6A-AS	0.4393514	1.50E-42
TRPV1	DENND6A-AS	0.4059886	5.19E-36
PKD2L2	DENND6A-AS	0.4893107	8.29E-54
TRPV2	LINC01215	0.404703	8.97E-36
MCOLN2	LINC01215	0.5297847	2.12E-64
TRPC5	CFAP44-AS1	0.5671272	1.57E-75
TRPV1	CFAP44-AS1	0.5463941	3.54E-69
PKD2L2	CFAP44-AS1	0.6825408	7.14E-121
PKD1L3	CFAP44-AS1	0.4535649	1.45E-45
TRPV2	LINC00877	0.491653	2.20E-54
MCOLN2	LINC00877	0.4821668	4.41E-52
TRPC5	ARHGEF3-AS	0.8940028	2.79E-306
TRPV1	ARHGEF3-AS	0.7073413	1.61E-133
PKD2L2	ARHGEF3-AS	0.6862597	1.09E-122
PKD1L3	ARHGEF3-AS	0.7820042	3.15E-181
TRPC5	MAGI1-IT1	0.5836912	6.11E-81
TRPV1	MAGI1-IT1	0.5798959	1.13E-79
PKD2L2	MAGI1-IT1	0.5871923	4.00E-82
PKD1L3	MAGI1-IT1	0.4534337	1.55E-45
TRPC5	RUVBL1-AS1	0.5697089	2.36E-76
TRPV1	RUVBL1-AS1	0.5453389	7.24E-69
PKD2L2	RUVBL1-AS1	0.4170243	4.30E-38
PKD1L3	RUVBL1-AS1	0.5260591	2.30E-63
TRPV4	ST3GAL6-AS	0.4464005	5.00E-44
TRPC5	MAGI1-AS1	0.6434702	2.56E-103
TRPV1	MAGI1-AS1	0.6575229	2.49E-109
PKD2L2	MAGI1-AS1	0.6246043	9.96E-96
PKD1L3	MAGI1-AS1	0.4933929	8.19E-55
TRPC5	TMEM108-AS	0.4276711	3.55E-40
TRPV1	TMEM108-AS	0.6543867	5.84E-108

PKD2L2	TMEM108-AS	0.5402071	2.29E-67
PKD1L3	TMEM108-AS	0.514855	2.51E-60
TRPC5	PDZRN3-AS1	0.492273	1.55E-54
TRPV1	PDZRN3-AS1	0.4658215	2.78E-48
PKD2L2	PDZRN3-AS1	0.5514509	1.10E-70
PKD1L3	PDZRN3-AS1	0.4759143	1.33E-50
TRPC5	SIDT1-AS1	0.4611268	3.15E-47
TRPV1	SIDT1-AS1	0.4251416	1.13E-39
PKD2L2	SIDT1-AS1	0.4465673	4.61E-44
TRPC5	IQCJ-SCHIP	0.7503494	6.24E-159
TRPV1	IQCJ-SCHIP	0.770554	9.48E-173
PKD2L2	IQCJ-SCHIP	0.625917	3.07E-96
PKD1L3	IQCJ-SCHIP	0.766469	7.64E-170
PKD2L2	NAALADL2-A	0.4921	1.71E-54
TRPC5	TERC	0.5139915	4.26E-60
TRPV1	TERC	0.6081162	1.65E-89
PKD2L2	TERC	0.5874803	3.19E-82
PKD1L3	TERC	0.4573386	2.17E-46
PKD2	AADACL2-AS	0.4134185	2.10E-37
TRPC5	TBL1XR1-AS	0.5032919	2.63E-57
TRPV1	TBL1XR1-AS	0.5243032	7.01E-63
PKD2L2	TBL1XR1-AS	0.6285507	2.84E-97
PKD1L3	TBL1XR1-AS	0.4890878	9.39E-54
TRPC5	RASA2-IT1	0.6325258	7.51E-99
TRPV1	RASA2-IT1	0.6572276	3.36E-109
PKD2L2	RASA2-IT1	0.6706225	3.12E-115
PKD1L3	RASA2-IT1	0.6565973	6.35E-109
TRPC5	WWTR1-IT1	0.5665788	2.34E-75
TRPV1	WWTR1-IT1	0.5536283	2.41E-71
PKD2L2	WWTR1-IT1	0.4863586	4.33E-53
PKD1L3	WWTR1-IT1	0.5310688	9.26E-65
PKD2	ADAMTS9-AS	0.5590139	5.43E-73
TRPC5	LINC02066	0.5053836	7.62E-58
TRPV1	LINC02066	0.4042318	1.10E-35
PKD2L2	LINC02066	0.4906317	3.93E-54
PKD1L3	LINC02066	0.4083833	1.86E-36
PKD2L2	OPA1-AS1	0.4264643	6.16E-40
TRPC5	PRICKLE2-A	0.7017129	1.56E-130
TRPV1	PRICKLE2-A	0.5760122	2.15E-78
PKD2L2	PRICKLE2-A	0.5602961	2.18E-73
PKD1L3	PRICKLE2-A	0.6003896	1.02E-86
TRPC5	TPRG1-AS2	0.5165778	8.72E-61
PKD1L3	TPRG1-AS2	0.4093679	1.22E-36
PKD2L2	ABCC5-AS1	0.4712369	1.61E-49
TRPV1	IGF2BP2-AS	0.5063362	4.32E-58
PKD1L3	IGF2BP2-AS	0.4406698	8.00E-43
TRPC5	LPP-AS1	0.699475	2.29E-129
TRPV1	LPP-AS1	0.6853669	3.00E-122
PKD2L2	LPP-AS1	0.6862745	1.07E-122
PKD1L3	LPP-AS1	0.7447166	2.59E-155
TRPC5	ETV5-AS1	0.5583858	8.48E-73

TRPV1	ETV5-AS1	0.5264863	1.75E-63
PKD2L2	ETV5-AS1	0.4516811	3.71E-45
PKD1L3	ETV5-AS1	0.575196	3.97E-78
TRPV2	XXYLT1-AS2	0.4235097	2.36E-39
MCOLN2	XXYLT1-AS2	0.505039	9.34E-58
TRPC5	THOC7-AS1	0.5298025	2.10E-64
TRPV1	THOC7-AS1	0.4579795	1.57E-46
PKD2L2	THOC7-AS1	0.5474479	1.72E-69
PKD1L3	THOC7-AS1	0.5103097	3.99E-59
TRPC5	PSMD6-AS2	0.4086293	1.68E-36
PKD1L3	PSMD6-AS2	0.4337753	2.09E-41
TRPC5	ZBTB20-AS1	0.6207575	3.04E-94
TRPV1	ZBTB20-AS1	0.5472263	2.00E-69
PKD2L2	ZBTB20-AS1	0.684996	4.55E-122
PKD1L3	ZBTB20-AS1	0.6414649	1.74E-102
TRPC5	ZBTB20-AS5	0.7819477	3.47E-181
TRPV1	ZBTB20-AS5	0.6794401	2.22E-119
PKD2L2	ZBTB20-AS5	0.7218946	1.40E-141
PKD1L3	ZBTB20-AS5	0.7457725	5.52E-156
TRPC5	ZBTB20-AS3	0.7139324	4.16E-137
TRPV1	ZBTB20-AS3	0.5907612	2.40E-83
PKD2L2	ZBTB20-AS3	0.7318352	2.18E-147
PKD1L3	ZBTB20-AS3	0.6261253	2.55E-96
TRPC5	ZBTB20-AS4	0.4089209	1.48E-36
TRPV1	MYLK-AS2	0.5593893	4.16E-73
PKD1L3	MYLK-AS2	0.5649301	7.76E-75
TRPC5	GLYCTK-AS1	0.6016041	3.76E-87
TRPV1	GLYCTK-AS1	0.5434955	2.52E-68
PKD2L2	GLYCTK-AS1	0.490619	3.96E-54
PKD1L3	GLYCTK-AS1	0.5236302	1.07E-62
TRPV1	RHOA-IT1	0.462166	1.85E-47
TRPC5	PEX5L-AS1	0.6758674	1.11E-117
PKD1L3	PEX5L-AS1	0.5571984	1.97E-72
TRPC5	PEX5L-AS2	0.4875006	2.29E-53
PKD1L3	PEX5L-AS2	0.4180163	2.77E-38
TRPV1	FLNB-AS1	0.4321182	4.54E-41
TRPC5	VIPR1-AS1	0.4688996	5.55E-49
TRPV1	VIPR1-AS1	0.4827865	3.14E-52
TRPC6	FGF12-AS2	0.4396291	1.32E-42
TRPC6	FGF12-AS3	0.4803898	1.17E-51
TRPC5	LARS2-AS1	0.7483865	1.17E-157
TRPV1	LARS2-AS1	0.669631	8.94E-115
PKD2L2	LARS2-AS1	0.6426921	5.39E-103
PKD1L3	LARS2-AS1	0.6556948	1.57E-108
TRPC5	ITGB5-AS1	0.5400016	2.62E-67
TRPV1	ITGB5-AS1	0.513022	7.70E-60
PKD2L2	ITGB5-AS1	0.5348772	7.76E-66
PKD1L3	ITGB5-AS1	0.5075635	2.08E-58
TRPC5	STX18-IT1	0.6780644	1.01E-118
TRPV1	STX18-IT1	0.7176339	3.62E-139
PKD2L2	STX18-IT1	0.6205251	3.73E-94

PKD1L3	STX18-IT1	0.6896232	2.36E-124
TRPV4	LINC02473	0.4763314	1.06E-50
TRPM6	LINC02473	0.4985711	4.16E-56
TRPC5	KCNIP4-IT1	0.404892	8.28E-36
PKD2L2	KCNIP4-IT1	0.5449718	9.29E-69
PKD2L2	CPEB2-DT	0.4154791	8.50E-38
PKD1	CTBP1-AS	0.4244841	1.52E-39
TRPC5	LNX1-AS1	0.6845004	7.95E-122
TRPV1	LNX1-AS1	0.4838452	1.75E-52
PKD2L2	LNX1-AS1	0.5583615	8.63E-73
PKD1L3	LNX1-AS1	0.5628042	3.61E-74
TRPC6	LINC02428	0.4387419	2.01E-42
TRPC5	GSTCD-AS1	0.4886601	1.19E-53
TRPV1	GSTCD-AS1	0.4444057	1.32E-43
PKD2L2	GSTCD-AS1	0.5327234	3.16E-65
TRPC5	PARM1-AS1	0.4205718	8.86E-39
PKD1L3	PARM1-AS1	0.4324845	3.83E-41
TRPC5	ARHGEF38-I	0.4806417	1.02E-51
TRPA1	SLC7A11-AS	0.5613232	1.05E-73
TRPC4	LINC02266	0.4586489	1.12E-46
TRPV2	LINC02273	0.4827616	3.18E-52
MCOLN2	LINC02273	0.4644686	5.62E-48
TRPV2	LINC02362	0.4091775	1.32E-36
TRPM3	LINC01612	0.6419138	1.13E-102
PKD2	HAND2-AS1	0.4992741	2.76E-56
TRPV6	LINC01093	0.4317128	5.48E-41
TRPC5	TET2-AS1	0.6237085	2.22E-95
TRPV1	TET2-AS1	0.520154	9.50E-62
PKD2L2	TET2-AS1	0.7140493	3.58E-137
PKD1L3	TET2-AS1	0.5640409	1.48E-74
TRPV2	IL21-AS1	0.4608066	3.71E-47
MCOLN2	IL21-AS1	0.5920884	8.37E-84
TRPC5	SMAD1-AS2	0.7180557	2.10E-139
TRPV1	SMAD1-AS2	0.7811708	1.36E-180
PKD2L2	SMAD1-AS2	0.6684937	2.98E-114
PKD1L3	SMAD1-AS2	0.7423312	8.25E-154
TRPV1	CYP2U1-AS1	0.4241697	1.75E-39
PKD2L2	CYP2U1-AS1	0.5260135	2.37E-63
PKD1L3	CYP2U1-AS1	0.4700399	3.04E-49
TRPC5	SDAD1-AS1	0.4366171	5.50E-42
TRPM5	LINC01019	0.5035549	2.25E-57
TRPM3	GDNF-AS1	0.4426128	3.14E-43
PKD2L2	VCAN-AS1	0.4491376	1.31E-44
TRPC5	CTD-2350J1	0.4924728	1.38E-54
TRPV1	CTD-2350J1	0.5692336	3.35E-76
PKD2L2	CTD-2350J1	0.4979544	5.95E-56
TRPC5	MAST4-IT1	0.5340413	1.34E-65
TRPV1	MAST4-IT1	0.4525905	2.36E-45
PKD2L2	MAST4-IT1	0.6880882	1.37E-123
PKD1L3	MAST4-IT1	0.4249718	1.22E-39
TRPC5	TH2LCRR	0.4358253	7.99E-42

TRPV1	TH2LCRR	0.5708967	9.80E-77
PKD2L2	TH2LCRR	0.5988059	3.72E-86
PKD1L3	TH2LCRR	0.419691	1.31E-38
TRPM3	LINC02058	0.4841534	1.47E-52
TRPM1	SMAD5-AS1	0.8957988	0.00E+00
TRPV1	CXXC5-AS1	0.4654925	3.30E-48
MCOLN2	MIR3142HG	0.4839288	1.67E-52
TRPC5	SAP30L-AS1	0.449792	9.45E-45
TRPV1	SAP30L-AS1	0.44706	3.62E-44
PKD2L2	SAP30L-AS1	0.4211537	6.82E-39
TRPC5	ZNF346-IT1	0.4660305	2.49E-48
TRPV1	ZNF346-IT1	0.4828186	3.08E-52
PKD2L2	ZNF346-IT1	0.4573762	2.13E-46
TRPC5	CARMN	0.5543699	1.44E-71
TRPV1	CARMN	0.6531886	1.93E-107
PKD2L2	CARMN	0.4487491	1.58E-44
PKD1L3	CARMN	0.6159421	2.05E-92
TRPC5	SLIT3-AS1	0.5347284	8.55E-66
TRPV1	SLIT3-AS1	0.7571946	1.85E-163
PKD2L2	SLIT3-AS1	0.4808929	8.88E-52
PKD1L3	SLIT3-AS1	0.6784812	6.38E-119
TRPC5	SEMA5A-AS1	0.5415127	9.56E-68
TRPV1	SEMA5A-AS1	0.6361502	2.61E-100
PKD2L2	SEMA5A-AS1	0.6962638	1.04E-127
PKD1L3	SEMA5A-AS1	0.5430343	3.44E-68
MCOLN3	FAM153CP	0.4046724	9.08E-36
TRPC5	CLMAT3	0.605534	1.44E-88
TRPV1	CLMAT3	0.6289732	1.94E-97
PKD2L2	CLMAT3	0.4620169	1.99E-47
PKD1L3	CLMAT3	0.6069255	4.50E-89
TRPM7	NADK2-AS1	0.431541	5.94E-41
PKD2L2	NADK2-AS1	0.462712	1.39E-47
TRPC5	LINC02863	0.7559128	1.34E-162
TRPV1	LINC02863	0.5849189	2.36E-81
PKD2L2	LINC02863	0.6549949	3.18E-108
PKD1L3	LINC02863	0.7175174	4.21E-139
TRPC5	DIAPH1-AS1	0.6979169	1.47E-128
TRPV1	DIAPH1-AS1	0.6099735	3.43E-90
PKD2L2	DIAPH1-AS1	0.7531213	9.53E-161
PKD1L3	DIAPH1-AS1	0.6673985	9.43E-114
TRPV1	NBAT1	0.4343629	1.59E-41
PKD2L2	NBAT1	0.4215216	5.78E-39
PKD1L3	NBAT1	0.4202562	1.02E-38
TRPC5	E2F3-IT1	0.6911032	4.29E-125
TRPV1	E2F3-IT1	0.7263417	3.80E-144
PKD2L2	E2F3-IT1	0.620912	2.65E-94
PKD1L3	E2F3-IT1	0.7245208	4.34E-143
TRPC5	ZNF451-AS1	0.4718565	1.16E-49
TRPV1	ZNF451-AS1	0.6404222	4.69E-102
PKD2L2	ZNF451-AS1	0.4806517	1.01E-51
PKD1L3	ZNF451-AS1	0.5664781	2.52E-75

TRPC5	BTBD9-AS1	0.6491431	1.05E-105
TRPV1	BTBD9-AS1	0.6358714	3.38E-100
PKD2L2	BTBD9-AS1	0.5709751	9.25E-77
PKD1L3	BTBD9-AS1	0.6202213	4.88E-94
TRPV2	HLA-DQB1-A	0.4442266	1.44E-43
MCOLN2	HLA-DQB1-A	0.4053609	6.78E-36
TRPC5	RUNX2-AS1	0.4556792	5.02E-46
TRPV1	RUNX2-AS1	0.4598642	6.01E-47
PKD1L3	RUNX2-AS1	0.4438753	1.71E-43
TRPC5	LAMA4-AS1	0.4021667	2.62E-35
TRPV1	LAMA4-AS1	0.4169653	4.41E-38
PKD1L3	LAMA4-AS1	0.4680992	8.45E-49
TRPV4	BVES-AS1	0.4018637	2.98E-35
TRPM6	BVES-AS1	0.4014	3.62E-35
TRPV2	WAKMAR2	0.4284432	2.49E-40
MCOLN2	WAKMAR2	0.539036	4.99E-67
PKD2	MAP3K4-AS1	0.4491686	1.29E-44
TRPV2	LINC02528	0.4061907	4.76E-36
MCOLN2	LINC02528	0.4798894	1.54E-51
TRPV1	ARMC2-AS1	0.4027577	2.04E-35
TRPV2	LINC01624	0.431787	5.30E-41
TRPC5	PTPRK-AS1	0.8134714	1.89E-207
TRPV1	PTPRK-AS1	0.6545779	4.82E-108
PKD2L2	PTPRK-AS1	0.5803697	7.86E-80
PKD1L3	PTPRK-AS1	0.7884742	2.99E-186
TRPC5	REV3L-IT1	0.4871883	2.73E-53
TRPV1	REV3L-IT1	0.5184967	2.66E-61
PKD2L2	REV3L-IT1	0.4459199	6.32E-44
PKD1L3	REV3L-IT1	0.4824667	3.74E-52
TRPM8	MLIP-IT1	0.4158836	7.11E-38
TRPC5	PHACTR2-AS	0.5229965	1.60E-62
TRPV1	PHACTR2-AS	0.6111615	1.25E-90
PKD2L2	PHACTR2-AS	0.5028978	3.31E-57
PKD1L3	PHACTR2-AS	0.6129133	2.79E-91
TRPV2	LINC01010	0.4077809	2.41E-36
PKD1L3	MAP3K5-AS1	0.4078735	2.32E-36
TRPC5	AIRN	0.6657681	5.21E-113
TRPV1	AIRN	0.5608074	1.51E-73
PKD2L2	AIRN	0.6647912	1.44E-112
PKD1L3	AIRN	0.6379859	4.67E-101
TRPC5	SNX9-AS1	0.5454261	6.83E-69
TRPV1	SNX9-AS1	0.5351145	6.64E-66
PKD2L2	SNX9-AS1	0.7133148	9.11E-137
PKD1L3	SNX9-AS1	0.4744337	2.94E-50
TRPC5	SYNJ2-IT1	0.5538128	2.12E-71
TRPV1	SYNJ2-IT1	0.4744365	2.93E-50
PKD1L3	SYNJ2-IT1	0.5142236	3.70E-60
TRPV2	PSMB8-AS1	0.4756436	1.53E-50
MCOLN2	PSMB8-AS1	0.4602569	4.91E-47
PKD2L2	MYB-AS1	0.4747561	2.47E-50
TRPV2	PRKAR1B-AS	0.7159417	3.20E-138

MCOLN2	PRKAR1B-AS	0.464068	6.91E-48
PKD2L2	MACC1-AS1	0.512214	1.26E-59
TRPC5	LINC02860	0.5157496	1.45E-60
TRPV1	LINC02860	0.4582122	1.39E-46
PKD2L2	LINC02860	0.4535912	1.43E-45
PKD1L3	LINC02860	0.5229453	1.65E-62
TRPV2	TRG-AS1	0.6192658	1.13E-93
MCOLN2	TRG-AS1	0.6375997	6.72E-101
TRPC6	POU6F2-AS2	0.5010544	9.78E-57
TRPV4	DLX6-AS1	0.5037268	2.03E-57
TRPM6	DLX6-AS1	0.4714819	1.42E-49
TRPC5	DOCK4-AS1	0.6028034	1.40E-87
TRPV1	DOCK4-AS1	0.5602698	2.22E-73
PKD2L2	DOCK4-AS1	0.6953144	3.17E-127
PKD1L3	DOCK4-AS1	0.5674242	1.26E-75
TRPC5	SND1-IT1	0.5603886	2.04E-73
TRPV1	SND1-IT1	0.6813231	2.77E-120
PKD2L2	SND1-IT1	0.6398325	8.19E-102
PKD1L3	SND1-IT1	0.6496422	6.42E-106
TRPV2	PTPRN2-AS1	0.5297929	2.11E-64
TRPC5	PRKAR2B-AS	0.4587137	1.08E-46
TRPV1	PRKAR2B-AS	0.4613066	2.87E-47
PKD2L2	PRKAR2B-AS	0.5677758	9.76E-76
TRPC5	ST7-OT4	0.5980435	6.93E-86
TRPV1	ST7-OT4	0.6655945	6.24E-113
PKD2L2	ST7-OT4	0.7058444	1.02E-132
PKD1L3	ST7-OT4	0.6303195	5.68E-98
TRPC5	ST7-AS2	0.486915	3.18E-53
TRPV1	ST7-AS2	0.598594	4.43E-86
PKD2L2	ST7-AS2	0.6881056	1.34E-123
PKD1L3	ST7-AS2	0.5281646	6.00E-64
TRPC5	RNF216-IT1	0.5851306	2.00E-81
TRPV1	RNF216-IT1	0.6573426	2.99E-109
PKD2L2	RNF216-IT1	0.5387535	6.02E-67
PKD1L3	RNF216-IT1	0.6349861	7.71E-100
TRPV2	TRBV11-2	0.5250626	4.33E-63
MCOLN2	TRBV11-2	0.531971	5.16E-65
MCOLN2	CARD11-AS1	0.4022562	2.52E-35
TRPC5	CREB3L2-AS	0.6133922	1.85E-91
TRPV1	CREB3L2-AS	0.6835491	2.31E-121
PKD2L2	CREB3L2-AS	0.6844093	8.81E-122
PKD1L3	CREB3L2-AS	0.663575	5.09E-112
TRPC1	MAGI2-AS3	0.5183774	2.87E-61
PKD2	MAGI2-AS3	0.7387222	1.44E-151
PKD2	KMT2E-AS1	-0.417847	2.98E-38
TRPV2	LINC00996	0.5328833	2.85E-65
MCOLN2	LINC00996	0.6091514	6.89E-90
TRPC5	WWC3-AS1	0.700073	1.12E-129
TRPV1	WWC3-AS1	0.7542459	1.72E-161
PKD2L2	WWC3-AS1	0.5952815	6.48E-85
PKD1L3	WWC3-AS1	0.7439376	8.05E-155

PKD2L2	EIF1AX-AS1	0.4517569	3.57E-45
TRPV1	INE1	0.4111403	5.66E-37
TRPV2	MIR223HG	0.4116465	4.54E-37
MCOLN2	MIR223HG	0.4228054	3.24E-39
TRPC5	DIAPH2-AS1	0.4784772	3.31E-51
TRPV1	DIAPH2-AS1	0.5928725	4.48E-84
PKD2L2	DIAPH2-AS1	0.5215577	3.95E-62
PKD1L3	DIAPH2-AS1	0.5522597	6.25E-71
TRPC5	FTX	0.5682806	6.74E-76
TRPV1	FTX	0.6110414	1.38E-90
PKD2L2	FTX	0.6706053	3.18E-115
PKD1L3	FTX	0.5392184	4.42E-67
TRPV2	LINC01281	0.4483125	1.96E-44
MCOLN2	LINC01281	0.5250068	4.49E-63
TRPV4	PLS3-AS1	0.4799767	1.46E-51
TRPC5	XIAP-AS1	0.7786873	1.01E-178
TRPV1	XIAP-AS1	0.7310139	6.74E-147
PKD2L2	XIAP-AS1	0.6032486	9.65E-88
PKD1L3	XIAP-AS1	0.7494037	2.57E-158
TRPC5	DANT2	0.735236	1.96E-149
TRPV1	DANT2	0.7572375	1.73E-163
PKD2L2	DANT2	0.7011381	3.11E-130
PKD1L3	DANT2	0.6895954	2.43E-124
TRPV1	LINC00894	0.4740299	3.65E-50
PKD2L2	LINC00894	0.4089649	1.45E-36
TRPV1	ALG13-AS1	0.4922973	1.53E-54
TRPV2	LINC00892	0.4602414	4.95E-47
MCOLN2	LINC00892	0.474488	2.85E-50
TRPC5	TMLHE-AS1	0.4566758	3.03E-46
TRPV1	TMLHE-AS1	0.4221722	4.32E-39
PKD2L2	TMLHE-AS1	0.4181838	2.57E-38
PKD1L3	TMLHE-AS1	0.455567	5.31E-46
TRPC5	KDM5C-IT1	0.7460705	3.57E-156
TRPV1	KDM5C-IT1	0.7511674	1.83E-159
PKD2L2	KDM5C-IT1	0.5960208	3.57E-85
PKD1L3	KDM5C-IT1	0.7399303	2.59E-152
TRPC5	TCEAL3-AS1	0.4891664	8.99E-54
TRPV1	TCEAL3-AS1	0.5638854	1.65E-74
PKD2L2	TCEAL3-AS1	0.5740408	9.44E-78
PKD1L3	TCEAL3-AS1	0.4311648	7.07E-41
TRPC5	ZNF630-AS1	0.6346427	1.06E-99
TRPV1	ZNF630-AS1	0.6858175	1.80E-122
PKD2L2	ZNF630-AS1	0.5404902	1.89E-67
PKD1L3	ZNF630-AS1	0.582559	1.46E-80
TRPM7	XIST	0.4134114	2.11E-37
TRPC5	LINC00102	0.6822559	9.81E-121
TRPV1	LINC00102	0.6708866	2.35E-115
PKD2L2	LINC00102	0.5374753	1.40E-66
PKD1L3	LINC00102	0.6341998	1.60E-99
PKD2	MSC-AS1	0.5150646	2.21E-60
TRPV2	LINC00861	0.442478	3.35E-43

MCOLN2	LINC00861	0.5613303	1.04E-73
TRPC6	LINC01414	0.6116957	7.92E-91
PKD1L3	LACTB2-AS1	0.4094394	1.18E-36
TRPM7	LINC00824	0.5693324	3.11E-76
TRPV1	MAL2-AS1	0.4239672	1.92E-39
PKD2L2	MAL2-AS1	0.4525377	2.42E-45
TRPM5	LINC01608	0.7428569	3.86E-154
TRPC5	ASAP1-IT2	0.4735292	4.77E-50
TRPV1	ASAP1-IT2	0.4819908	4.86E-52
PKD2L2	ASAP1-IT2	0.4550491	6.89E-46
PKD1L3	ASAP1-IT2	0.5038376	1.90E-57
TRPC5	TMEM75	0.4960209	1.82E-55
TRPV1	TMEM75	0.5814732	3.38E-80
PKD2L2	TMEM75	0.496133	1.70E-55
TRPM3	CASC9	0.4036336	1.41E-35
PKD2L2	INTS9-AS1	0.4417917	4.67E-43
TRPC6	ZFPM2-AS1	0.5158443	1.37E-60
TRPC5	CARNMT1-AS	0.445015	9.82E-44
TRPV1	CARNMT1-AS	0.5039202	1.81E-57
PKD2L2	CARNMT1-AS	0.4925439	1.33E-54
PKD1L3	CARNMT1-AS	0.4247625	1.34E-39
TRPV1	PCA3	0.5643695	1.17E-74
PKD1L3	PCA3	0.4460549	5.92E-44
TRPC5	GNA14-AS1	0.6192498	1.15E-93
PKD1L3	GNA14-AS1	0.503986	1.74E-57
TRPC5	LINC00484	0.470252	2.72E-49
TRPV1	LINC00484	0.5909157	2.13E-83
PKD2L2	LINC00484	0.466487	1.97E-48
PKD1L3	LINC00484	0.5239142	8.96E-63
TRPV1	HSD17B3-AS	0.426896	5.06E-40
PKD1L3	HSD17B3-AS	0.4310246	7.55E-41
TRPM3	LINC01474	0.6723088	5.15E-116
PKD2	GAS1RR	0.4927814	1.16E-54
TRPV1	MAMDC2-AS1	0.4889496	1.02E-53
PKD2L2	MAMDC2-AS1	0.4057121	5.84E-36
PKD1L3	MAMDC2-AS1	0.5083008	1.34E-58
TRPV1	MIRLET7A1H	0.4250452	1.18E-39
PKD2L2	MIRLET7A1H	0.4818706	5.19E-52
TRPM1	LINC02247	0.4969963	1.04E-55
TRPV1	RMRP	0.4516848	3.70E-45
PKD2L2	RMRP	0.483651	1.95E-52
PKD2	ARRDC1-AS1	-0.410357	7.95E-37
TRPC5	GSN-AS1	0.5548829	1.00E-71
TRPV1	GSN-AS1	0.7076175	1.14E-133
PKD2L2	GSN-AS1	0.5345069	9.89E-66
PKD1L3	GSN-AS1	0.6694715	1.06E-114
TRPV2	C9orf139	0.5334515	1.97E-65
MCOLN2	C9orf139	0.467753	1.01E-48
TRPC5	ASTN2-AS1	0.7528125	1.52E-160
TRPV1	ASTN2-AS1	0.6272585	9.17E-97
PKD2L2	ASTN2-AS1	0.6544778	5.33E-108

PKD1L3	ASTN2-AS1	0.6339262	2.06E-99
TRPC5	UBE2R2-AS1	0.5917973	1.06E-83
TRPV1	UBE2R2-AS1	0.5958137	4.22E-85
PKD2L2	UBE2R2-AS1	0.5194355	1.49E-61
PKD1L3	UBE2R2-AS1	0.6160009	1.95E-92
TRPV1	MIR1302-9H	0.4313748	6.42E-41
TRPM1	LINC01451	0.461044	3.28E-47
TRPC5	KCNQ1OT1	0.6744303	5.26E-117
TRPV1	KCNQ1OT1	0.6206354	3.38E-94
PKD2L2	KCNQ1OT1	0.6608502	8.43E-111
PKD1L3	KCNQ1OT1	0.6134739	1.73E-91
TRPC5	FAR1-IT1	0.6389548	1.88E-101
TRPV1	FAR1-IT1	0.6637199	4.38E-112
PKD2L2	FAR1-IT1	0.4743809	3.02E-50
PKD1L3	FAR1-IT1	0.574288	7.85E-78
TRPC5	PTPRJ-AS1	0.8178652	1.65E-211
TRPV1	PTPRJ-AS1	0.734066	9.99E-149
PKD2L2	PTPRJ-AS1	0.6792271	2.81E-119
PKD1L3	PTPRJ-AS1	0.7894613	4.94E-187
TRPC5	MADD-AS1	0.6306231	4.31E-98
TRPV1	MADD-AS1	0.6468196	1.01E-104
PKD2L2	MADD-AS1	0.559697	3.34E-73
PKD1L3	MADD-AS1	0.6258555	3.24E-96
TRPC5	MALAT1	0.8301893	1.68E-223
TRPV1	MALAT1	0.6710221	2.04E-115
PKD2L2	MALAT1	0.6499712	4.64E-106
PKD1L3	MALAT1	0.7019775	1.13E-130
TRPV1	NEAT1	0.4890692	9.49E-54
TRPC5	RSF1-IT2	0.4329086	3.14E-41
PKD2L2	RSF1-IT2	0.4490501	1.36E-44
TRPC5	SCARNA9	0.428743	2.17E-40
PKD2L2	SCARNA9	0.6453514	4.19E-104
PKD1L3	SCARNA9	0.4122391	3.51E-37
TRPC5	ARAP1-AS2	0.5964039	2.62E-85
TRPV1	ARAP1-AS2	0.6394928	1.13E-101
PKD2L2	ARAP1-AS2	0.5364432	2.77E-66
PKD1L3	ARAP1-AS2	0.6066686	5.58E-89
PKD2	MIR100HG	0.5718313	4.90E-77
TRPV6	PPP1R14B-A	0.457795	1.72E-46
TRPC5	CARS1-AS1	0.6481972	2.64E-105
TRPV1	CARS1-AS1	0.648327	2.33E-105
PKD2L2	CARS1-AS1	0.5866528	6.11E-82
PKD1L3	CARS1-AS1	0.5720319	4.22E-77
TRPC5	SHANK2-AS1	0.5768519	1.14E-78
TRPV1	SHANK2-AS1	0.4598159	6.16E-47
PKD2L2	SHANK2-AS1	0.4924223	1.42E-54
PKD1L3	SHANK2-AS1	0.5079027	1.70E-58
TRPC5	SHANK2-AS2	0.7290708	9.55E-146
TRPV1	SHANK2-AS2	0.6315719	1.81E-98
PKD2L2	SHANK2-AS2	0.5931383	3.62E-84
PKD1L3	SHANK2-AS2	0.6497056	6.03E-106

TRPC5	SHANK2-AS3	0.4132739	2.24E-37
PKD1L3	SHANK2-AS3	0.4075596	2.65E-36
TRPC5	APOA1-AS	0.6841119	1.23E-121
TRPV1	APOA1-AS	0.5651232	6.75E-75
PKD2L2	APOA1-AS	0.4238354	2.04E-39
PKD1L3	APOA1-AS	0.5855588	1.43E-81
TRPC5	ALKBH3-AS1	0.4003739	5.56E-35
PKD2	LINC00702	0.4622217	1.79E-47
MCOLN2	LINC02648	0.4424895	3.33E-43
PKD2L2	LINC02676	0.4177322	3.14E-38
TRPV1	LINC02669	0.4685548	6.65E-49
PKD1L3	CELF2-AS1	0.4090998	1.37E-36
TRPV4	LINC02630	0.4028412	1.97E-35
PKD2	ACTA2-AS1	0.4256335	9.00E-40
TRPM7	ENTPD1-AS1	0.4164945	5.43E-38
PKD2	EMX2OS	0.4740315	3.64E-50
TRPC5	FAM53B-AS1	0.643878	1.73E-103
TRPV1	FAM53B-AS1	0.7001844	9.78E-130
PKD2L2	FAM53B-AS1	0.5985339	4.65E-86
PKD1L3	FAM53B-AS1	0.6757923	1.20E-117
TRPV1	ITPRIP-AS1	0.4271648	4.47E-40
PKD1L3	ITPRIP-AS1	0.4178188	3.02E-38
TRPC5	EDRF1-AS1	0.5122842	1.21E-59
TRPV1	EDRF1-AS1	0.5929694	4.14E-84
PKD2L2	EDRF1-AS1	0.4769442	7.61E-51
PKD1L3	EDRF1-AS1	0.4938984	6.14E-55
TRPC5	GRK5-IT1	0.6502704	3.46E-106
TRPV1	GRK5-IT1	0.6061232	8.81E-89
PKD2L2	GRK5-IT1	0.5972033	1.37E-85
PKD1L3	GRK5-IT1	0.6429188	4.34E-103
TRPV2	IATPR	0.4522677	2.77E-45
MCOLN2	IATPR	0.5013867	8.05E-57
TRPV3	LINC02881	0.4192906	1.57E-38
TRPC5	PLCE1-AS2	0.4366262	5.48E-42
TRPV1	PLCE1-AS2	0.4391704	1.64E-42
PKD2L2	PLCE1-AS2	0.5882929	1.69E-82
PKD1L3	PLCE1-AS2	0.4381399	2.67E-42
TRPC5	KCNMA1-AS3	0.560694	1.64E-73
TRPC5	DIP2C-AS1	0.5293824	2.75E-64
TRPV1	DIP2C-AS1	0.6137492	1.36E-91
PKD2L2	DIP2C-AS1	0.5025196	4.14E-57
PKD1L3	DIP2C-AS1	0.6026967	1.52E-87
TRPV2	LINC02446	0.4118028	4.25E-37
MCOLN2	LINC02446	0.506708	3.46E-58
TRPC5	RERG-AS1	0.5769428	1.06E-78
TRPV1	RERG-AS1	0.5531788	3.30E-71
PKD2L2	RERG-AS1	0.5247375	5.33E-63
PKD1L3	RERG-AS1	0.420029	1.13E-38
MCOLN2	LINC02390	0.4812023	7.49E-52
MCOLN2	PARP11-AS1	0.4348232	1.28E-41
TRPV2	LINC02416	0.4696946	3.65E-49

MCOLN2	LINC02416	0.6101959	2.84E-90
MCOLN2	LINC02422	0.4855679	6.73E-53
TRPV1	SOX5-AS1	0.4963849	1.47E-55
PKD2L2	SOX5-AS1	0.4301305	1.14E-40
MCOLN2	IFNG-AS1	0.4816709	5.79E-52
TRPV1	LRP1-AS	0.5400029	2.62E-67
PKD1L3	LRP1-AS	0.4985959	4.10E-56
TRPV2	LINC02391	0.4122938	3.43E-37
TRPM3	LINC02418	0.5292329	3.02E-64
MCOLN2	LINC00943	0.4838341	1.76E-52
MCOLN2	LINC02413	0.4542505	1.03E-45
TRPV2	LINC02384	0.536109	3.45E-66
MCOLN2	LINC02384	0.5816597	2.93E-80
TRPV2	USP30-AS1	0.5196709	1.28E-61
MCOLN2	USP30-AS1	0.4682491	7.81E-49
TRPM3	LINC02463	0.5471079	2.17E-69
TRPV6	KRT7-AS	0.4133641	2.15E-37
TRPV2	PCED1B-AS1	0.4324928	3.81E-41
PKD2	TBX5-AS1	0.5428543	3.88E-68
MCOLN2	LINC02397	0.4237052	2.16E-39
TRPC5	CLIP1-AS1	0.7502951	6.77E-159
TRPV1	CLIP1-AS1	0.6710616	1.95E-115
PKD2L2	CLIP1-AS1	0.5702409	1.59E-76
PKD1L3	CLIP1-AS1	0.6283544	3.40E-97
TRPC5	CACNA1C-AS	0.6068106	4.95E-89
TRPV1	CACNA1C-AS	0.575017	4.54E-78
PKD2L2	CACNA1C-AS	0.5985093	4.74E-86
PKD1L3	CACNA1C-AS	0.6011168	5.61E-87
TRPC5	CACNA1C-IT	0.6560044	1.15E-108
TRPV1	CACNA1C-IT	0.6170065	8.13E-93
PKD2L2	CACNA1C-IT	0.5817012	2.83E-80
PKD1L3	CACNA1C-IT	0.6478092	3.86E-105
TRPC5	STARD13-AS	0.7138964	4.35E-137
TRPV1	STARD13-AS	0.6236981	2.24E-95
PKD2L2	STARD13-AS	0.7297227	3.93E-146
PKD1L3	STARD13-AS	0.6126043	3.64E-91
TRPC4	LINC02334	0.8835773	1.48E-289
TRPC5	LINC00457	0.6190187	1.40E-93
TRPV1	LINC00457	0.4489108	1.46E-44
PKD1L3	LINC00457	0.4860498	5.15E-53
TRPC5	STARD13-IT	0.6200702	5.57E-94
TRPV1	STARD13-IT	0.5606173	1.73E-73
PKD2L2	STARD13-IT	0.7083211	4.78E-134
PKD1L3	STARD13-IT	0.5348785	7.75E-66
TRPC5	SPATA13-AS	0.8692358	5.33E-269
TRPV1	SPATA13-AS	0.7006496	5.60E-130
PKD2L2	SPATA13-AS	0.6717144	9.73E-116
PKD1L3	SPATA13-AS	0.7337762	1.49E-148
MCOLN2	LINC00402	0.4635337	9.12E-48
TRPV2	LINC00426	0.4822645	4.18E-52
MCOLN2	LINC00426	0.5306708	1.20E-64

TRPV4	LINC00392	0.4467259	4.27E-44
TRPC5	ENOX1-AS1	0.4717067	1.26E-49
TRPV1	ENOX1-AS1	0.5176384	4.53E-61
PKD2L2	ENOX1-AS1	0.5160799	1.18E-60
PKD1L3	ENOX1-AS1	0.4879101	1.82E-53
TRPC5	PCCA-AS1	0.7542705	1.66E-161
TRPV1	PCCA-AS1	0.5891499	8.59E-83
PKD2L2	PCCA-AS1	0.6428482	4.65E-103
PKD1L3	PCCA-AS1	0.6495225	7.22E-106
TRPC5	FGF14-IT1	0.4442002	1.46E-43
PKD2L2	FGF14-IT1	0.4218133	5.07E-39
TRPC5	LINC02344	0.5182863	3.03E-61
TRPV1	LINC02344	0.5051914	8.54E-58
PKD2L2	LINC02344	0.6840854	1.27E-121
PKD1L3	LINC02344	0.4407124	7.83E-43
TRPM1	SOX21-AS1	0.4682833	7.67E-49
TRPM1	LINC00462	0.5780296	4.67E-79
TRPC5	N4BP2L2-IT	0.4228437	3.19E-39
TRPV1	N4BP2L2-IT	0.4797963	1.62E-51
PKD2L2	N4BP2L2-IT	0.5054455	7.34E-58
TRPC5	FRY-AS1	0.4177907	3.06E-38
TRPC5	MYCBP2-AS2	0.4187044	2.04E-38
TRPV1	MYCBP2-AS2	0.4557465	4.85E-46
PKD2L2	MYCBP2-AS2	0.5010602	9.74E-57
PKD1L3	MYCBP2-AS2	0.4568068	2.84E-46
TRPC5	ATP11A-AS1	0.5190031	1.94E-61
TRPV1	ATP11A-AS1	0.6785903	5.66E-119
PKD2L2	ATP11A-AS1	0.543995	1.80E-68
PKD1L3	ATP11A-AS1	0.6471067	7.64E-105
TRPC5	LINC00517	0.628517	2.93E-97
TRPV1	LINC00517	0.4942095	5.14E-55
PKD2L2	LINC00517	0.6060249	9.56E-89
PKD1L3	LINC00517	0.4861556	4.85E-53
TRPV2	LINC01146	0.4098647	9.83E-37
TRPV2	LINC02328	0.5279285	6.98E-64
MCOLN2	LINC02328	0.5108164	2.94E-59
TRPV2	LINC02325	0.4622664	1.75E-47
MCOLN2	LINC02325	0.5513955	1.14E-70
TRPC5	ITPK1-AS1	0.6180656	3.23E-93
TRPV1	ITPK1-AS1	0.6767074	4.44E-118
PKD2L2	ITPK1-AS1	0.5646102	9.79E-75
PKD1L3	ITPK1-AS1	0.4889126	1.04E-53
PKD2	MEG3	0.429732	1.37E-40
TRPC5	MEG8	0.4289947	1.93E-40
PKD2L2	MEG8	0.5136621	5.21E-60
TRPV2	LINC02285	0.5306707	1.20E-64
TRPC5	LINC00216	0.4804162	1.15E-51
TRPV1	LINC00216	0.4883935	1.39E-53
PKD2L2	LINC00216	0.6138199	1.28E-91
PKD1L3	LINC00216	0.405753	5.74E-36
TRPV2	FAM30A	0.4467567	4.20E-44

MCOLN2	FAM30A	0.5009012	1.07E-56
PKD2L2	HIF1A-AS3	0.4158506	7.22E-38
TRPC5	LINC02250	0.8643103	1.70E-262
TRPV1	LINC02250	0.7297094	4.00E-146
PKD2L2	LINC02250	0.5745754	6.33E-78
PKD1L3	LINC02250	0.8245085	7.42E-218
TRPV2	LINC02694	0.4912066	2.84E-54
MCOLN2	LINC02694	0.5039662	1.76E-57
TRPC5	VPS39-DT	0.4639719	7.27E-48
TRPV1	VPS39-DT	0.4908622	3.45E-54
PKD2L2	VPS39-DT	0.4474484	2.99E-44
TRPM6	RYR3-DT	0.4017901	3.07E-35
TRPM7	GABPB1-AS1	0.5995539	2.02E-86
TRPC5	SALRNA2	0.7944583	4.68E-191
TRPV1	SALRNA2	0.7323537	1.07E-147
PKD2L2	SALRNA2	0.5328507	2.91E-65
PKD1L3	SALRNA2	0.766624	5.94E-170
TRPC5	TPM1-AS	0.7418934	1.55E-153
TRPV1	TPM1-AS	0.6066373	5.73E-89
PKD2L2	TPM1-AS	0.4567338	2.95E-46
PKD1L3	TPM1-AS	0.6699121	6.63E-115
TRPC5	INO80-AS1	0.5920178	8.85E-84
TRPV1	INO80-AS1	0.5232724	1.34E-62
PKD2L2	INO80-AS1	0.4699296	3.22E-49
PKD1L3	INO80-AS1	0.5123143	1.18E-59
TRPC5	NPTN-IT1	0.4473767	3.10E-44
TRPV1	NPTN-IT1	0.4901736	5.09E-54
TRPM7	NPTN-IT1	0.4543651	9.71E-46
PKD1L3	NPTN-IT1	0.4028373	1.97E-35
TRPC5	LINC02204	0.4304659	9.78E-41
TRPV1	LINC02204	0.4365458	5.69E-42
PKD1L3	LINC02204	0.4309845	7.69E-41
TRPC5	EHD4-AS1	0.6229991	4.17E-95
TRPV1	EHD4-AS1	0.6616547	3.69E-111
PKD2L2	EHD4-AS1	0.4855437	6.82E-53
PKD1L3	EHD4-AS1	0.6219501	1.06E-94
TRPM7	MIR4713HG	0.4851496	8.49E-53
TRPM7	RORA-AS1	0.4160982	6.47E-38
TRPM3	LINC01586	0.8482549	5.98E-243
TRPV2	LINC02244	0.4131321	2.38E-37
TRPC5	MGC15885	0.4565078	3.30E-46
TRPV1	MGC15885	0.4159578	6.88E-38
PKD1L3	MGC15885	0.4307034	8.76E-41
TRPC5	THSD4-AS1	0.8458119	3.56E-240
TRPV1	THSD4-AS1	0.6615171	4.25E-111
PKD2L2	THSD4-AS1	0.538955	5.26E-67
PKD1L3	THSD4-AS1	0.646845	9.85E-105
TRPC5	PCSK6-AS1	0.7137371	5.33E-137
TRPV1	PCSK6-AS1	0.5497064	3.66E-70
PKD2L2	PCSK6-AS1	0.5173387	5.45E-61
PKD1L3	PCSK6-AS1	0.57032	1.50E-76

TRPM7	OIP5-AS1	0.4123304	3.37E-37
TRPV6	IDH2-DT	0.4037615	1.34E-35
TRPV4	CEROX1	0.4546169	8.56E-46
TRPC5	CDR2-DT	0.6048357	2.58E-88
TRPV1	CDR2-DT	0.619851	6.75E-94
PKD2L2	CDR2-DT	0.5279744	6.78E-64
PKD1L3	CDR2-DT	0.5605179	1.86E-73
TRPC5	LCMT1-AS2	0.6899814	1.56E-124
TRPV1	LCMT1-AS2	0.7531627	8.95E-161
PKD2L2	LCMT1-AS2	0.6571286	3.71E-109
PKD1L3	LCMT1-AS2	0.6159702	2.00E-92
TRPV1	RSL1D1-DT	0.4894683	7.58E-54
TRPV4	LINC02188	0.4396052	1.33E-42
TRPV2	LINC02132	0.449529	1.08E-44
MCOLN2	LINC02132	0.5048495	1.05E-57
TRPV2	RRN3P2	0.4907803	3.62E-54
MCOLN2	RRN3P2	0.4644957	5.54E-48
TRPV2	CORO1A-AS1	0.4966461	1.27E-55
MCOLN2	CORO1A-AS1	0.5247868	5.16E-63
TRPV2	CYLD-AS1	0.5002251	1.59E-56
MCOLN2	CYLD-AS1	0.5959405	3.81E-85
TRPM1	LINC02137	0.6641104	2.92E-112
PKD2	LINC02185	0.4258766	8.06E-40
TRPC5	SMCR5	0.8481056	8.87E-243
TRPV1	SMCR5	0.8130633	4.45E-207
PKD2L2	SMCR5	0.6773128	2.29E-118
PKD1L3	SMCR5	0.7703987	1.23E-172
TRPV2	CCL3-AS1	0.4537229	1.34E-45
MCOLN2	CCL3-AS1	0.5185681	2.55E-61
TRPC5	THRA1/BTR	0.4670083	1.50E-48
PKD2L2	THRA1/BTR	0.5053466	7.79E-58
PKD1L3	THRA1/BTR	0.4201884	1.05E-38
TRPM1	MIR2117HG	0.4097657	1.03E-36
TRPC5	RAI1-AS1	0.8767357	1.98E-279
TRPV1	RAI1-AS1	0.8078312	2.14E-202
PKD2L2	RAI1-AS1	0.6364467	1.98E-100
PKD1L3	RAI1-AS1	0.793766	1.72E-190
PKD1L3	SKAP1-AS1	0.4138285	1.75E-37
PKD2L2	ABCA9-AS1	0.4885615	1.26E-53
TRPV4	LINC00511	0.4690876	5.03E-49
MCOLN2	TSPOAP1-AS	0.4305167	9.55E-41
PKD2L2	PRKCA-AS1	0.4399076	1.15E-42
PKD2	MIR22HG	0.426121	7.21E-40
TRPV4	LINC01152	0.4653955	3.47E-48
TRPV4	ROCR	0.4787711	2.82E-51
TRPV4	SOX9-AS1	0.5340115	1.37E-65
TRPC5	MPRIP-AS1	0.7013985	2.27E-130
TRPV1	MPRIP-AS1	0.7973513	1.95E-193
PKD2L2	MPRIP-AS1	0.6445306	9.24E-104
PKD1L3	MPRIP-AS1	0.6627769	1.16E-111
PKD2	MYHAS	0.5579098	1.19E-72

TRPC5	EPN2-AS1	0.7739433	3.30E-175
TRPV1	EPN2-AS1	0.7330014	4.37E-148
PKD2L2	EPN2-AS1	0.6848485	5.37E-122
PKD1L3	EPN2-AS1	0.7034761	1.84E-131
TRPV2	FMNL1-DT	0.5761331	1.96E-78
MCOLN2	FMNL1-DT	0.5524328	5.55E-71
TRPC5	ST8SIA5-DT	0.6148059	5.48E-92
TRPV1	ST8SIA5-DT	0.5229416	1.66E-62
PKD2L2	ST8SIA5-DT	0.5343543	1.09E-65
PKD1L3	ST8SIA5-DT	0.5723026	3.45E-77
TRPV2	L3MBTL4-AS	0.4412548	6.04E-43
TRPC5	SNHG22	0.5185608	2.56E-61
TRPV1	SNHG22	0.6082137	1.52E-89
PKD2L2	SNHG22	0.6096296	4.59E-90
PKD1L3	SNHG22	0.5397903	3.02E-67
TRPV1	LINC01544	0.4347758	1.31E-41
TRPC6	LINC01901	0.770511	1.02E-172
TRPC5	MIR133A1HG	0.5312004	8.50E-65
TRPV1	MIR133A1HG	0.5640098	1.51E-74
PKD2L2	MIR133A1HG	0.7436883	1.16E-154
PKD1L3	MIR133A1HG	0.488822	1.09E-53
PKD2	LINC01415	0.465111	4.02E-48
TRPC6	DSG1-AS1	0.4288258	2.09E-40
MCOLN2	SIRPG-AS1	0.4823574	3.97E-52
MCOLN2	LINC01726	0.4218089	5.08E-39
TRPV2	LINC01727	0.4037647	1.33E-35
MCOLN2	LINC01727	0.4222829	4.11E-39
TRPC5	DTD1-AS1	0.651266	1.30E-106
TRPV1	DTD1-AS1	0.6135977	1.55E-91
PKD2L2	DTD1-AS1	0.587804	2.48E-82
PKD1L3	DTD1-AS1	0.6345022	1.21E-99
TRPC5	ISM1-AS1	0.4870381	2.96E-53
TRPV1	ISM1-AS1	0.6995811	2.02E-129
PKD2L2	ISM1-AS1	0.5042975	1.45E-57
PKD1L3	ISM1-AS1	0.5795411	1.48E-79
TRPC5	MACROD2-AS	0.4245711	1.46E-39
TRPM1	FAM182A	0.4952108	2.89E-55
TRPC5	PTPRT-AS1	0.5990133	3.14E-86
TRPV1	PTPRT-AS1	0.4159394	6.94E-38
PKD2L2	PTPRT-AS1	0.4871748	2.75E-53
PKD1L3	PTPRT-AS1	0.4433011	2.25E-43
TRPV2	PELATON	0.4619498	2.06E-47
MCOLN2	RBM38-AS1	0.4713488	1.52E-49
TRPV1	LINC01429	0.5513791	1.15E-70
PKD1L3	LINC01429	0.4949296	3.40E-55
TRPM3	APCDD1L-DT	0.5951088	7.45E-85
TRPC6	LINC01749	0.6080068	1.81E-89
TRPC5	ITCH-IT1	0.599265	2.56E-86
TRPV1	ITCH-IT1	0.6318638	1.38E-98
PKD2L2	ITCH-IT1	0.7041439	8.15E-132
PKD1L3	ITCH-IT1	0.5264646	1.78E-63

TRPV2	CEP250-AS1	0.5874348	3.31E-82
MCOLN2	CEP250-AS1	0.5020538	5.44E-57
PKD2L2	DLGAP4-AS1	0.4331135	2.85E-41
PKD1L3	DLGAP4-AS1	0.4046924	9.01E-36
TRPC5	UBOX5-AS1	0.4036032	1.43E-35
TRPV1	UBOX5-AS1	0.5029758	3.16E-57
TRPC5	BCL2L1-AS1	0.6880558	1.42E-123
TRPV1	BCL2L1-AS1	0.6887163	6.67E-124
PKD2L2	BCL2L1-AS1	0.5932468	3.32E-84
PKD1L3	BCL2L1-AS1	0.5700912	1.78E-76
TRPC5	KIZ-AS1	0.6382177	3.76E-101
TRPV1	KIZ-AS1	0.5964627	2.50E-85
PKD2L2	KIZ-AS1	0.6290139	1.87E-97
PKD1L3	KIZ-AS1	0.580252	8.60E-80
TRPV1	SLC24A3-AS	0.4700081	3.09E-49
PKD2L2	SLC24A3-AS	0.5743124	7.70E-78
PKD1L3	SLC24A3-AS	0.4279074	3.18E-40
TRPC5	GNA15-DT	0.4230289	2.93E-39
TRPV1	GNA15-DT	0.4136621	1.89E-37
PKD1L3	GNA15-DT	0.4841479	1.48E-52
TRPV2	CARD8-AS1	0.5295205	2.51E-64
MCOLN2	CARD8-AS1	0.470298	2.65E-49
TRPM1	LINC02560	0.4213989	6.11E-39
