# Supplementary material for: Construction and validation of a transient receptor potential-related long noncoding RNA signature for prognosis prediction in breast cancer patients
Source: Medicine (Baltimore). 2023 Nov 17;102(46):e35978. doi: 10.1097/MD.0000000000035978 (PMC10659707; doi:10.1097/MD.0000000000035978)
Supplement: Supplementary file 3 [file medi-102-e35978-s003.pdf]

Table S3. The prognosis related lncRNAs in breast cancer

id	HR	HR.95L	HR.95H	pvalue
TNFRSF14-A	0.7250555	0.6191187	0.849119	6.62E-05
CAMTA1-AS1	0.7879344	0.6454709	0.9618413	0.0191646
RERE-AS1	0.7301315	0.6171239	0.8638331	0.0002463
PIK3CD-AS2	0.8212806	0.7095966	0.9505428	0.0082883
LINC01786	0.8333543	0.7003866	0.9915658	0.039834
NFIA-AS2	0.855654	0.7415776	0.9872788	0.0327337
PKN2-AS1	1.219305	1.0360461	1.4349792	0.0170278
VAV3-AS1	0.7959968	0.6806887	0.930838	0.0042686
LRIG2-DT	0.781693	0.6311773	0.9681019	0.0240054
LINC02609	0.7891588	0.6570299	0.9478588	0.0113173
LINC00623	0.8490617	0.7333464	0.9830359	0.0286081
LINC01765	0.7164703	0.5670537	0.9052578	0.0052047
LINC00624	1.2475049	1.0930748	1.4237528	0.0010386
LRRC8D-DT	0.8484979	0.7207977	0.9988221	0.0483674
LINC01633	1.2050483	1.0562734	1.3747779	0.0055327
KIAA1614-A	1.2644887	1.0461561	1.5283873	0.0152433
MIR205HG	0.9135737	0.8481245	0.9840736	0.0171601
LINC01353	0.8095577	0.669845	0.9784108	0.0288323
TGFB2-AS1	0.8881787	0.7942741	0.9931853	0.0375346
LINC01341	0.8610555	0.7468155	0.9927707	0.0394109
IPO9-AS1	0.7757259	0.6087062	0.9885734	0.0400858
KCNH1-IT1	0.8572426	0.7718589	0.9520716	0.0040088
LINC01344	0.8856674	0.7967773	0.9844744	0.0244536
RABGAP1L-I	0.7165384	0.5463146	0.9398014	0.0160137
OVAAL	0.8233004	0.6814078	0.9947399	0.0439455
LINC01036	1.1622601	1.0291952	1.312529	0.0153575
LINC00582	0.8503134	0.7400429	0.9770147	0.0221321
PINK1-AS	1.4500991	1.0398448	2.0222128	0.0285073
ATP1A1-AS1	0.684481	0.5124198	0.9143172	0.0102765
THBS3-AS1	0.7905627	0.6515641	0.9592138	0.0172173
CHRM3-AS2	0.8595818	0.7638175	0.9673526	0.012048
LRRC8C-DT	0.8105922	0.6870227	0.9563874	0.0128326
GORAB-AS1	1.2013677	1.0210559	1.4135215	0.027029
MRPL20-DT	1.3667244	1.1089763	1.6843782	0.0033888
PEF1-AS1	0.7603315	0.6314129	0.9155722	0.0038468
LAMTOR5-AS	1.4674183	1.0824476	1.9893032	0.0135009
AKT3-IT1	0.7403228	0.5709029	0.9600195	0.0233477
TTLL10-AS1	0.8279559	0.690569	0.9926756	0.0414128
ECE1-AS1	0.714866	0.5783332	0.8836313	0.0019091
TDRKH-AS1	1.3541606	1.0654017	1.7211827	0.0132234
C2CD4D-AS1	1.2956185	1.0689916	1.5702904	0.0082896
TFAP2E-AS1	0.7953516	0.6562541	0.9639317	0.0195697
ID2-AS1	1.2467942	1.0231309	1.5193517	0.0287675
FLJ31356	1.2207769	1.0335983	1.4418526	0.0188186
PPP1R21-DT	1.2428353	1.0387736	1.4869838	0.0175172
LINC02245	0.8318191	0.7086058	0.9764569	0.0243706
PGM5P4-AS1	0.7009177	0.5551446	0.8849688	0.0028154
C2orf91	0.8228972	0.7014958	0.9653083	0.0166878
FSIP2-AS1	0.8174132	0.6911925	0.9666834	0.018477

ANKRD44-AS	0.7686108	0.6628551	0.8912394	0.0004931
ANKRD44-IT	0.7499653	0.6004051	0.9367809	0.0112308
LINC01854	1.214855	1.0382705	1.4214723	0.0151569
KIAA2012-A	0.8139994	0.7124825	0.9299806	0.0024612
LINC01856	0.8850395	0.7858728	0.9967199	0.0439936
C2orf49-DT	1.4745813	1.0920849	1.9910447	0.0112473
LINC01827	0.7928298	0.643972	0.976097	0.0286706
LINC01117	0.9015525	0.8183266	0.9932426	0.0359787
CATIP-AS1	0.775189	0.6512166	0.922762	0.0041827
SNED1-AS1	1.212245	1.0155537	1.4470312	0.0331032
LINC01918	0.8699417	0.757769	0.9987195	0.0479111
LINC00471	1.3639757	1.0530882	1.7666417	0.0186781
NIFK-AS1	0.5679719	0.3531118	0.9135694	0.0196621
HDAC4-AS1	0.786956	0.6402224	0.9673196	0.0228732
LINC01960	0.8265433	0.6839181	0.9989117	0.0486974
BOLA3-AS1	1.1991432	1.0063509	1.4288699	0.0422798
DGUOK-AS1	1.4279419	1.1188289	1.8224575	0.0042089
HOXD-AS2	0.8143656	0.7023554	0.944239	0.0065292
MFF-DT	1.7945313	1.3189378	2.4416183	0.0001976
LMCD1-AS1	1.2753018	1.0261104	1.5850095	0.0283555
LINC02084	0.8044223	0.6912888	0.9360707	0.0048889
SLC6A1-AS1	1.2386563	1.0047792	1.5269718	0.0450037
SRGAP3-AS2	0.8619984	0.7648024	0.9715467	0.0149798
FGD5-AS1	1.5668155	1.1222568	2.187477	0.0083542
LINC01267	0.8274187	0.6851829	0.999181	0.0490131
GOLGA4-AS1	1.5171712	1.1634119	1.9784982	0.0020885
ESRG	0.7711966	0.623958	0.9531799	0.0162357
CYB561D2	0.8191092	0.7011348	0.9569341	0.0119116
FOXP1-AS1	0.8262589	0.6873876	0.993186	0.0420737
ADAMTS9-AS	0.8712331	0.7622248	0.995831	0.0432569
LINC02018	0.8015227	0.655809	0.9796123	0.0306817
SEMA3F-AS1	0.6848339	0.5205713	0.9009285	0.0068187
LINC01215	0.8974558	0.8108938	0.9932582	0.0365564
SAMMSON	1.240124	1.0846927	1.4178279	0.0016337
SYNPR-AS1	0.8872238	0.8082719	0.9738876	0.011856
SIDT1-AS1	0.8555099	0.734916	0.9958923	0.0441083
LINC00881	0.7898444	0.6476026	0.9633286	0.0198723
STAG1-DT	1.2931154	1.0780395	1.5511003	0.0056126
NCK1-DT	1.5172077	1.1036988	2.0856407	0.0102376
ERICH6-AS1	0.7352864	0.574694	0.9407548	0.0144564
OPA1-AS1	1.2441102	1.029554	1.5033794	0.0237263
TPRG1-AS2	0.8369753	0.726772	0.9638892	0.0134905
SIAH2-AS1	0.7906489	0.6945715	0.9000162	0.00038
ACAP2-IT1	1.2324702	1.0098931	1.5041025	0.0397018
LINC01213	1.134548	1.0003455	1.2867546	0.0493746
LINC00885	1.1725827	1.0238605	1.3429078	0.0214073
LINC02037	1.1796773	1.0200957	1.3642236	0.0258628
ATP13A5-AS	1.1984129	1.0416834	1.3787235	0.0113727
PLCH1-AS1	1.2564773	1.0217967	1.545058	0.0304356
TIPARP-AS1	0.7693856	0.6241246	0.9484551	0.0140622
ARIH2OS	0.7020257	0.5088859	0.9684687	0.0311513

SCAANT1	0.7749887	0.6361583	0.9441165	0.0113761
ZBTB20-AS1	0.833769	0.7016513	0.9907638	0.0388869
MME-AS1	0.7313299	0.5406777	0.9892094	0.0423194
LINC02054	0.795187	0.6564748	0.963209	0.0191177
WNT5A-AS1	0.8644317	0.7690006	0.9717055	0.0146522
BSN-DT	1.1571701	1.0123532	1.3227029	0.0323591
LINC02043	1.2010204	1.040424	1.386406	0.0123824
SEMA3B-AS1	0.8419962	0.7432349	0.9538809	0.0068984
FGF12-AS2	1.2031843	1.018466	1.4214046	0.0296203
ITPR1-DT	1.2922954	1.022375	1.6334784	0.0319466
EGOT	0.8626623	0.7757182	0.9593511	0.0064188
ITGB5-AS1	0.8110799	0.6752467	0.9742375	0.0251519
THUMPD3-AS	1.5486223	1.1418581	2.1002882	0.0049044
LINC02482	0.8015647	0.6472241	0.9927102	0.0426611
LINC02265	0.7984889	0.6783	0.9399743	0.0068572
LINC02428	0.8666511	0.761973	0.9857095	0.0293228
FLJ20021	0.7598024	0.6061339	0.9524293	0.0171847
WDFY3-AS2	1.2758639	1.0165051	1.6013975	0.0356277
LNX1-AS2	1.2855633	1.0803849	1.5297075	0.0046333
LINC02263	0.7795467	0.6148572	0.9883483	0.0397097
SLC7A11-AS	1.2623862	1.0980195	1.4513575	0.0010612
LINC02466	1.1547621	1.0117623	1.317973	0.0328977
LINC02267	1.2239914	1.0521454	1.4239048	0.0088317
HHIP-AS1	0.7625031	0.6429627	0.9042685	0.0018295
LINC02275	1.1648004	1.0281955	1.3195544	0.0165373
LINC01098	1.19313	1.0112494	1.4077231	0.0363912
LINC02477	1.1785005	1.0047843	1.3822504	0.0435262
FAM160A1-D	1.2219179	1.0174879	1.4674213	0.0319082
UNC5C-AS1	0.8706945	0.760763	0.9965113	0.0443556
LINC02434	1.187364	1.0065599	1.4006451	0.0415961
USP46-DT	1.3706114	1.020613	1.8406346	0.0361185
HAND2-AS1	0.8564719	0.7374708	0.9946754	0.0423654
SRD5A3-AS1	1.3426593	1.0600729	1.7005754	0.0145331
LEF1-AS1	0.7677119	0.6324762	0.9318636	0.0075008
MIR4458HG	0.875152	0.7684846	0.996625	0.0443316
PURPL	1.1704601	1.0559711	1.2973622	0.0027271
LINC01513	0.7867884	0.6640855	0.9321632	0.005571
NNT-AS1	1.2961118	1.0128714	1.6585578	0.0392438
LINC02223	1.2387129	1.0533682	1.4566697	0.0096341
OSMR-AS1	0.835466	0.7014956	0.9950219	0.0438074
MEF2C-AS1	0.8248824	0.7098191	0.9585978	0.0120181
LINC02062	0.7813305	0.6260938	0.9750573	0.029001
LINC00992	1.07338	1.0008017	1.1512218	0.0474347
SNHG18	0.8549024	0.7447017	0.9814107	0.0259835
LINC01950	1.1866212	1.0302778	1.3666896	0.0176076
LINC01023	0.8213938	0.6757384	0.9984451	0.0482013
LINC02208	1.1688308	1.0254686	1.3322354	0.0194568
ARHGAP26-A	0.7770585	0.644212	0.9373001	0.0083676
KCNIP1-OT1	0.7985277	0.6419719	0.9932623	0.0433126
LINC02227	0.7997343	0.6477354	0.9874015	0.0377234
LINC01574	1.1976728	1.0539773	1.3609594	0.0056724

LINC01863	0.8527458	0.7674503	0.9475212	0.0030516
LINC02234	1.2717763	1.0757594	1.5035101	0.0048771
PRR7-AS1	1.2187	1.0324287	1.4385785	0.0194357
PP7080	1.4979352	1.0746287	2.0879862	0.0170918
SNHG4	1.1737642	1.0102254	1.3637773	0.0363618
LINC02236	1.359496	1.1481669	1.6097219	0.0003669
ZNF474-AS1	0.7960348	0.6647407	0.953261	0.0131214
P4HA2-AS1	1.3113184	1.0578052	1.6255885	0.0134118
FABP6-AS1	1.2146493	1.0126194	1.4569868	0.0361643
LINC00491	1.1725859	1.0456502	1.314931	0.0064577
HCG18	1.671823	1.1251399	2.4841286	0.010974
ELOVL2-AS1	0.8913994	0.8241919	0.9640873	0.0040479
LINC01016	0.9174042	0.8628248	0.9754362	0.0058749
BTBD9-AS1	1.2438119	1.0098511	1.5319763	0.0401533
LINC00472	0.8799334	0.7901817	0.9798794	0.0197923
MLIP-IT1	1.1968866	1.0214704	1.4024268	0.0262372
C6orf99	1.255699	1.049482	1.5024365	0.01286
STXBP5-AS1	1.5761937	1.268107	1.95913	4.12E-05
LINC01010	0.8466104	0.7354903	0.9745189	0.0203674
TAGAP-AS1	1.4318684	1.054497	1.9442892	0.0214518
LINC02532	1.1221816	1.0311748	1.2212202	0.0075541
LINC00602	1.1609464	1.0183572	1.3235008	0.0256128
MPC1-DT	1.3019146	1.0503242	1.6137699	0.0160357
SYNJ2-IT1	1.3347069	1.0656551	1.6716877	0.0119509
AFDN-DT	1.2363528	1.0417229	1.4673462	0.0151956
SDK1-AS1	1.2101032	1.0147524	1.4430611	0.0337545
PRKAR1B-AS	0.7702565	0.6234302	0.9516624	0.015558
LINC02888	1.1086708	1.0138602	1.2123476	0.0237126
SEC61G-DT	1.2728805	1.0434245	1.5527955	0.0173548
WEE2-AS1	0.7028928	0.5516711	0.8955666	0.0043398
WDR86-AS1	0.8369368	0.733768	0.9546114	0.0080015
PRKAG2-AS1	0.7783278	0.6533303	0.9272402	0.0050206
SUGCT-AS1	1.2157723	1.0497116	1.4081033	0.0091226
PPP1R35-AS	1.336516	1.0640883	1.6786905	0.0126285
HERPUD2-AS	1.3518691	1.0060716	1.816521	0.0454871
ST7-AS1	0.7218259	0.5687083	0.9161684	0.0073681
ST7-OT4	1.3045417	1.0541993	1.6143333	0.0144658
CRPPA-AS1	1.3295683	1.0853579	1.6287271	0.005941
FKBP14-AS1	1.4142452	1.1276266	1.7737161	0.0027048
ELN-AS1	0.8826046	0.7892539	0.9869966	0.0285646
TP53TG1	0.7493805	0.6126195	0.9166718	0.0050122
MNX1-AS1	1.0884441	1.0073077	1.1761159	0.0320186
LINC00685	0.795555	0.6370443	0.9935068	0.0436511
LINC01456	1.1285862	1.0375142	1.2276524	0.0048346
LINC01283	1.5190116	1.2209626	1.8898173	0.0001758
ZNF674-AS1	1.3812554	1.0393805	1.8355806	0.0260021
USP27X-DT	1.3312052	1.0026464	1.7674301	0.0479017
LINC00630	1.5933001	1.2231524	2.0754609	0.0005538
FTX	1.4090459	1.0728956	1.8505161	0.0136653
FIRRE	1.1248566	1.0090929	1.2539009	0.0337269
RAP2C-AS1	1.3005791	1.0228921	1.6536505	0.0319793

LINC01278	1.5433804	1.1137639	2.1387146	0.0091262
UXT-AS1	1.5872964	1.2008915	2.0980327	0.0011699
TLR8-AS1	1.1412655	1.0012383	1.300876	0.047873
MORF4L2-AS	1.3283419	1.0477134	1.6841363	0.0190321
ARMCX5-GPR	1.4870235	1.0716652	2.0633672	0.0175917
LINC00968	1.1543449	1.0063054	1.3241627	0.0403912
LINC02605	0.8410393	0.7307664	0.9679524	0.0157697
OTUD6B-AS1	1.8724445	1.4250279	2.4603366	6.72E-06
LINC01592	1.2119078	1.042418	1.4089555	0.0124043
MIR3150BHG	1.1402554	1.0093892	1.2880882	0.0348398
RAD21-AS1	1.289883	1.0814369	1.538507	0.0046471
ZNNT1	1.2630974	1.0360377	1.5399198	0.0208811
FAM83A-AS1	1.1312277	1.0339388	1.237671	0.0072015
LINC00824	1.1951708	1.037602	1.3766677	0.0134474
BAALC-AS1	1.1888883	1.0144903	1.3932665	0.03254
WASHC5-AS1	1.2510544	1.0242192	1.5281272	0.0282057
MAL2-AS1	1.2048102	1.0216352	1.4208276	0.026805
LINC01151	1.2054472	1.0020329	1.4501549	0.0475351
ASAP1-IT2	0.7534568	0.6294237	0.9019316	0.0020377
NCRNA0025C	1.315039	1.0973792	1.5758704	0.0030118
RHPN1-AS1	1.2735389	1.035445	1.5663811	0.0220322
LINC00536	1.171135	1.0579911	1.2963788	0.0023082
LNCOC1	1.2543261	1.1028414	1.4266185	0.0005593
CASC9	1.0880863	1.0057336	1.1771822	0.0355234
MAFA-AS1	0.8227887	0.7186136	0.9420657	0.0047427
MIR31HG	0.8248678	0.7138413	0.9531626	0.0090454
PGM5-AS1	0.8438301	0.7273295	0.9789912	0.0250866
SMC5-DT	1.4251296	1.0755796	1.888279	0.0136087
LINC01508	1.0958943	1.0047828	1.1952676	0.0386679
BARX1-DT	1.1393994	1.0067959	1.2894679	0.0387104
UNQ6494	0.8419605	0.7214392	0.9826157	0.0290756
DBH-AS1	0.796038	0.6853364	0.924621	0.0028284
BNC2-AS1	0.7925824	0.6743618	0.931528	0.0047934
C9orf163	1.354963	1.0873136	1.6884962	0.0068199
LINC01235	1.2017123	1.080654	1.3363319	0.0006945
PAPPA-AS2	0.7491929	0.5952473	0.9429527	0.0138754
PTENP1-AS	0.7895939	0.6258865	0.9961209	0.0462889
PCAT7	1.1540582	1.0226975	1.3022916	0.0201262
NAV2-AS6	0.8576924	0.7490802	0.9820527	0.0262753
LINC02726	1.1990106	1.0082638	1.4258435	0.0400669
LINC00294	1.4395023	1.1043728	1.8763292	0.0070563
WT1-AS	1.0909708	1.0094253	1.1791038	0.028046
ZNRD2-AS1	1.4055869	1.0034782	1.9688267	0.0476857
RSF1-IT1	1.2784421	1.0965112	1.4905586	0.0017109
LINC01395	1.1877206	1.028873	1.3710926	0.0188472
SENCR	0.7862646	0.6379862	0.9690052	0.024117
GSEC	1.2808157	1.0201299	1.6081177	0.0330386
LINC02752	0.8310805	0.7077821	0.975858	0.0239316
KRTAP5-AS1	1.1295221	1.0038378	1.2709425	0.0430107
LINC01001	1.4267082	1.1013773	1.848137	0.007119
CENATAC-DT	1.4468163	1.146449	1.8258792	0.0018639

C11orf72	1.1735065	1.0092691	1.36447	0.0375343
LINC02685	1.2174801	1.0343094	1.4330894	0.0180072
ADARB2-AS1	1.2032215	1.1059509	1.3090473	1.70E-05
MANCR	1.2370994	1.0905903	1.4032905	0.0009384
ST8SIA6-AS	1.1014486	1.020924	1.1883245	0.0126106
RPP38-DT	1.2820146	1.0287982	1.5975548	0.0269078
UNC5B-AS1	0.8384154	0.71916	0.9774466	0.0243623
DLG5-AS1	0.6924542	0.5508251	0.8704992	0.0016446
ZNF503-AS1	1.1506925	1.0385394	1.2749571	0.0073028
LNCAROD	1.1356519	1.024201	1.2592306	0.0157915
LINC00844	0.8670428	0.752553	0.9989505	0.0483254
ANK3-DT	0.813275	0.7083955	0.9336821	0.0033455
ACTA2-AS1	0.8508416	0.7347141	0.985324	0.0309719
ENTPD1-AS1	1.3190464	1.0175386	1.7098944	0.0365038
NUTM2A-AS1	1.4930739	1.055792	2.1114667	0.023389
PDCD4-AS1	0.6560982	0.5111818	0.8420972	0.0009344
PITRM1-AS1	1.35474	1.0355761	1.77227	0.02676
SFTPD-AS1	0.7775095	0.6151301	0.9827531	0.0352461
NUTM2B-AS1	1.4539711	1.0677981	1.979805	0.0174798
LINC02652	1.1842653	1.0022843	1.3992879	0.0469481
WAC-AS1	2.1165622	1.4469431	3.0960688	0.0001116
LINC02617	1.1889204	1.0099982	1.3995388	0.0375698
LINC02449	1.2186306	1.0158226	1.4619291	0.0332545
HOTAIR	1.0966046	1.0175057	1.1818525	0.0157655
LINC02408	1.2937108	1.0817464	1.5472089	0.0047928
LINC01234	1.2065636	1.1065221	1.31565	2.12E-05
CAPS2-AS1	0.6677647	0.4944457	0.9018376	0.008442
ADGRD1-AS1	1.229348	1.0883239	1.3886459	0.0008955
LINC02823	0.8155637	0.7021757	0.9472617	0.0076001
USP30-AS1	0.8528754	0.7437681	0.9779883	0.0226874
LINC02463	1.2072704	1.0408281	1.400329	0.0128222
PCED1B-AS1	0.8431941	0.7146534	0.9948548	0.0432703
LINC00987	0.8031961	0.6651912	0.9698324	0.0227012
LINC01465	0.7169992	0.5403058	0.9514757	0.021193
DDN-AS1	1.2913293	1.0547062	1.5810388	0.0132972
CCND2-AS1	0.7742866	0.6172406	0.9712903	0.0269785
LINC01559	1.2090705	1.0780171	1.356056	0.0011814
LRRK2-DT	0.805094	0.6852128	0.9459489	0.0084025
LLPH-DT	1.1766817	1.0094815	1.3715752	0.0374663
CACNA1C-IT	1.5313102	1.1619174	2.0181392	0.0024825
LINC00544	0.7869864	0.6395797	0.9683666	0.0235904
LINC00393	1.1244089	1.0322286	1.2248211	0.0072143
LINC00377	0.6429701	0.4756582	0.8691336	0.0040782
LINC01232	1.2816872	1.0604346	1.5491026	0.010262
LINC01055	0.839113	0.7319261	0.9619969	0.0118829
PRECSIT	1.2181834	1.0469499	1.4174231	0.0106613
LINC00348	1.2078291	1.02642	1.4213003	0.0229655
FGF14-AS2	0.7925322	0.6766133	0.9283106	0.003952
LINC00460	0.8723376	0.7621946	0.998397	0.0473384
SLC25A30-A	0.7640985	0.648694	0.9000339	0.0012787
LINC00645	1.2489563	1.0353822	1.5065856	0.0201602

LINC00519	0.7984562	0.6721386	0.9485131	0.0104205
LINC02588	1.1185718	1.0167987	1.2305315	0.0213213
LINC02318	1.3774194	1.1259059	1.6851181	0.0018536
DIO3OS	0.8191651	0.7283787	0.9212672	0.0008739
PSMA3-AS1	0.6395455	0.413576	0.9889802	0.0444538
ACTN1-AS1	0.7275835	0.602311	0.8789109	0.0009711
LINC01269	1.1416728	1.0144656	1.2848309	0.0279315
HIF1A-AS3	1.1970978	1.0175608	1.4083119	0.0300111
PWRN1	1.2255985	1.008658	1.4891982	0.0406878
THBS1-AS1	0.9273945	0.8656835	0.9935047	0.0319174
TPM1-AS	0.8153593	0.7071023	0.9401904	0.0049773
IQCH-AS1	1.3978678	1.0002617	1.9535231	0.0498211
C15orf54	1.1851316	1.0104567	1.390002	0.0368132
LINC00926	0.6792924	0.5672345	0.8134876	2.62E-05
MIR4713HG	1.1312875	1.0112474	1.2655769	0.0311312
GPR176-DT	1.2215641	1.0117606	1.4748733	0.0373815
VPS33B-DT	1.5147249	1.1945563	1.920706	0.0006096
LINC02207	0.8411706	0.7083149	0.9989454	0.0486103
LINC00923	0.7881601	0.644671	0.9635865	0.0202475
OIP5-AS1	1.4005645	1.0223746	1.9186517	0.0359266
SPATA41	0.7724015	0.6591796	0.9050705	0.0014066
BCAR4	1.0943519	1.0012215	1.1961451	0.0469374
LINC02130	0.8788224	0.7885881	0.9793819	0.0194461
ITFG1-AS1	1.2355748	1.0201153	1.4965418	0.0304898
LINC02133	1.2051458	1.0187335	1.4256686	0.0295221
CRNDE	1.419737	1.0868862	1.854521	0.0101343
LINC01572	1.3581158	1.1222982	1.6434836	0.0016571
HCCAT5	1.2157208	1.0045557	1.4712745	0.0447861
LINC01569	0.7897935	0.6267691	0.995221	0.0454381
CORO1A-AS1	0.8372735	0.7262127	0.965319	0.0144413
CENPN-AS1	1.2789735	1.0427352	1.5687331	0.0181961
FAM157C	1.2184373	1.0292188	1.4424429	0.021766
CARHSP1-DT	0.7704441	0.6247211	0.9501586	0.0147727
KIF1C-AS1	0.7608037	0.6111821	0.9470538	0.0144106
LINC02091	0.7351154	0.616036	0.8772127	0.0006427
ZFP3-DT	1.2180781	1.0250315	1.4474816	0.0250397
LINC01563	0.8255522	0.6983873	0.9758718	0.0246953
RARA-AS1	0.7391047	0.5916264	0.9233459	0.0077614
SP2-AS1	1.4484977	1.1452939	1.8319713	0.0019877
LINC00671	0.7450641	0.6014626	0.922951	0.0070613
LINC00511	1.1531945	1.014553	1.3107817	0.0291801
CACNA1G-AS	0.8500658	0.7233086	0.9990367	0.0486482
LINC01978	1.1634276	1.0207998	1.3259836	0.0233001
RNF213-AS1	0.7401351	0.606068	0.9038589	0.003164
ERVE-1	1.1373297	1.0180058	1.27064	0.022874
MAPT-AS1	0.8275077	0.7654933	0.894546	1.90E-06
MAPT-IT1	0.8512735	0.7863352	0.9215746	6.97E-05
LINC00482	1.1279596	1.0025345	1.2690764	0.0452791
TMEM105	1.1223009	1.004218	1.2542689	0.0419344
PIK3R5-DT	1.2423047	1.0621509	1.4530148	0.0066423
LINC00670	1.3427438	1.0681934	1.6878599	0.0115631

SNHG16	1.3040314	1.0254705	1.658261	0.03038
LINC01973	0.7759187	0.6334837	0.9503794	0.0142145
FMNL1-DT	0.8146025	0.6992639	0.9489652	0.0084773
LHX1-DT	1.1191072	1.0284785	1.217722	0.0090102
BAIAP2-DT	0.7424049	0.5749383	0.9586507	0.0223871
RUNDC3A-AS	1.1716397	1.0344994	1.3269601	0.0126321
HID1-AS1	1.2914333	1.0559463	1.5794364	0.012776
MIR497HG	0.8124779	0.6625834	0.9962826	0.0459605
ARHGAP28-A	0.8148185	0.6843956	0.9700957	0.0213855
LINC01925	1.1843688	1.0192268	1.3762682	0.0272073
DLGAP1-AS1	0.7160597	0.5301955	0.9670801	0.0293852
PPP4R1-AS1	0.7868079	0.6461137	0.958139	0.017061
DLGAP1-AS5	0.9000481	0.8286677	0.977577	0.0124934
AQP4-AS1	0.8361395	0.7275184	0.9609781	0.011716
TTC39C-AS1	0.798888	0.6840001	0.9330731	0.0045909
SNHG22	0.7210912	0.5870696	0.8857085	0.0018281
LINC01630	1.2220199	1.007767	1.4818233	0.041487
NDUFV2-AS1	0.5483599	0.39679	0.7578282	0.0002728
LINC01901	1.176876	1.0113781	1.3694553	0.0351797
LINC01539	0.7665348	0.6160194	0.9538265	0.0171342
LINC00668	1.07638	1.0008656	1.1575919	0.0473363
GAPLINC	0.7645872	0.6021841	0.9707886	0.0275724
PCAT18	0.8708641	0.8085541	0.9379758	0.0002618
LIVAR	1.3299934	1.1833199	1.4948472	1.72E-06
GREB1L-DT	0.6719926	0.560372	0.8058468	1.79E-05
KDSR-DT	0.7011453	0.553734	0.8877994	0.0031963
LINC00654	1.270378	1.0857114	1.4864543	0.002826
STK4-AS1	1.5659388	1.2506329	1.9607389	9.25E-05
CFAP61-AS1	1.2533856	1.0780178	1.4572816	0.0033157
LINC01275	1.264921	1.0686036	1.4973048	0.0063133
LINC01271	1.1565574	1.0025688	1.3341977	0.0460258
C20orf197	0.7477453	0.6145196	0.9098539	0.0036891
LINC01747	1.2276978	1.0046924	1.5002025	0.0448845
LINC00266-	1.348172	1.1065489	1.6425554	0.0030298
LAMA5-AS1	0.8378725	0.7272965	0.96526	0.0143012
ZNF337-AS1	1.3352284	1.0301608	1.7306374	0.028926
LINC01711	0.8024444	0.6925934	0.9297185	0.0033881
FLJ16779	0.8990842	0.8086028	0.9996903	0.0493347
LINC00659	1.1446887	1.023016	1.2808325	0.0184315
NORAD	1.3703227	1.0109258	1.8574896	0.0423584
DPP9-AS1	0.7933294	0.6557571	0.9597631	0.0171901
KLF2-DT	0.7165518	0.5455642	0.9411294	0.0165679
LINC00662	1.4398233	1.0702736	1.9369729	0.0160077
DPY19L3-DT	1.2370801	1.0114054	1.5131096	0.0384221
IGFL2-AS1	1.1549058	1.0359935	1.2874669	0.0093823

ivariate Cox regression on OS
