# Supplementary figures and images for: Construction and validation of a transient receptor potential-related long noncoding RNA signature for prognosis prediction in breast cancer patients
Source: Medicine (Baltimore). 2023 Nov 17;102(46):e35978. doi: 10.1097/MD.0000000000035978 (PMC10659707; doi:10.1097/MD.0000000000035978)

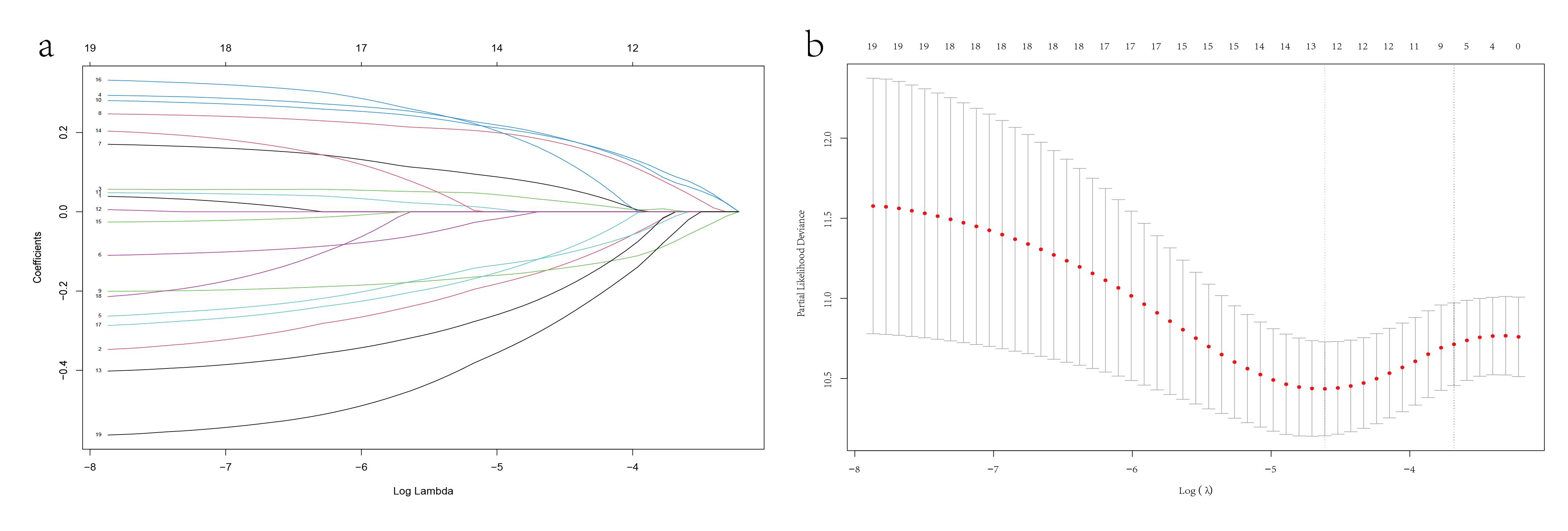

Supplement: Supplementary file 4 [file medi-102-e35978-s004.tif]

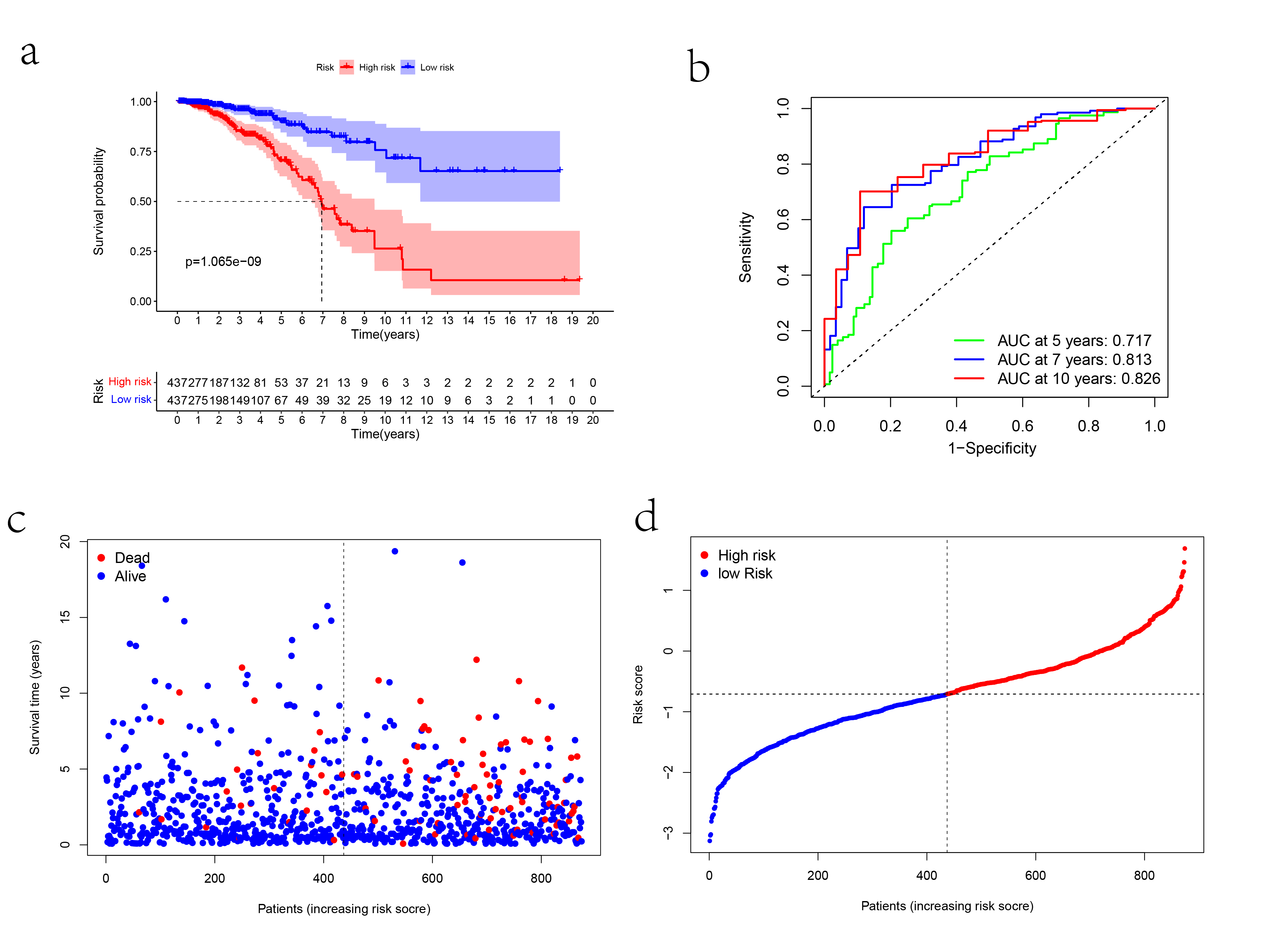

Supplement: Supplementary file 5 [file medi-102-e35978-s005.tif]

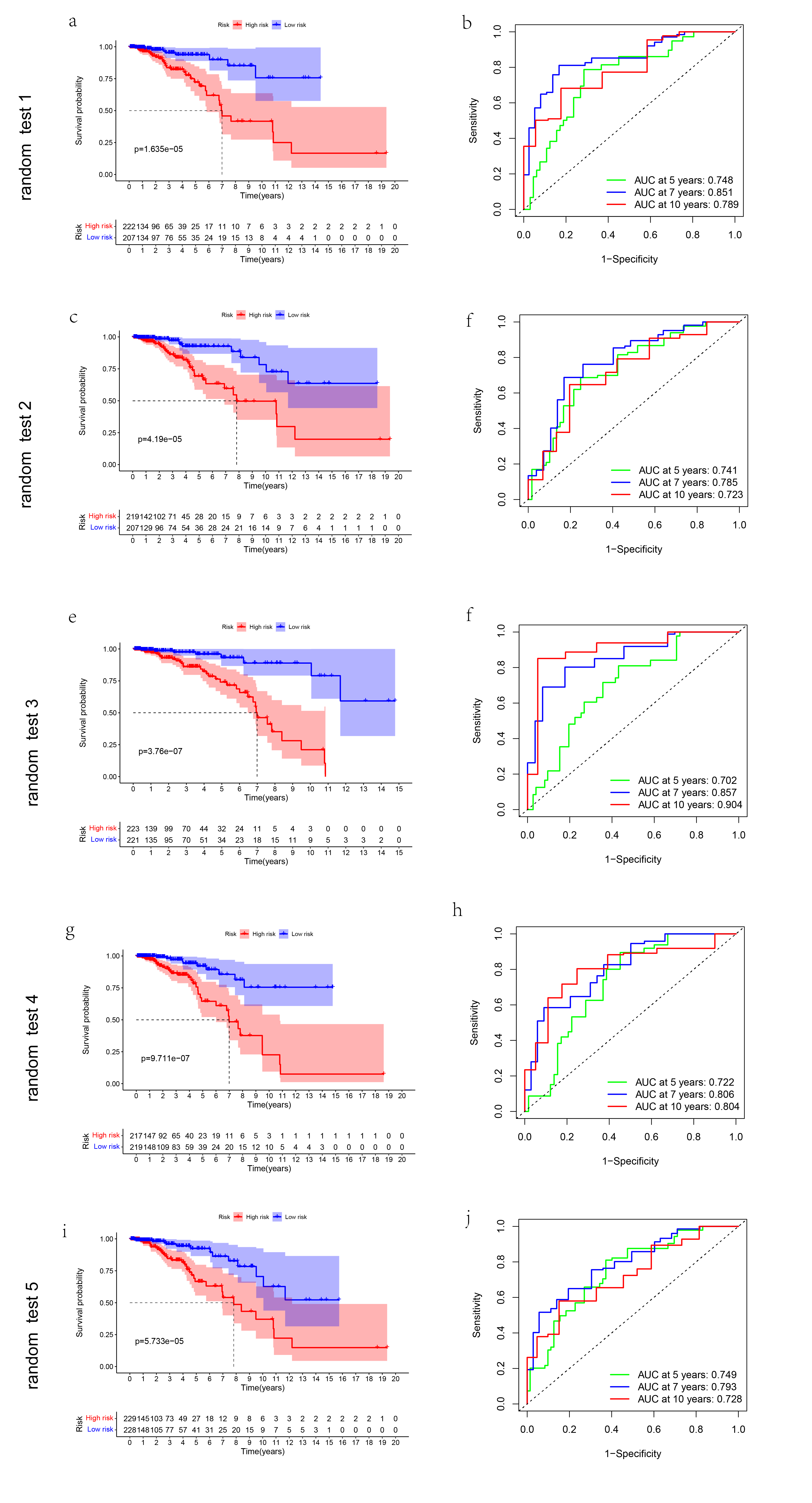

Supplement: Supplementary file 6 [file medi-102-e35978-s006.tif]

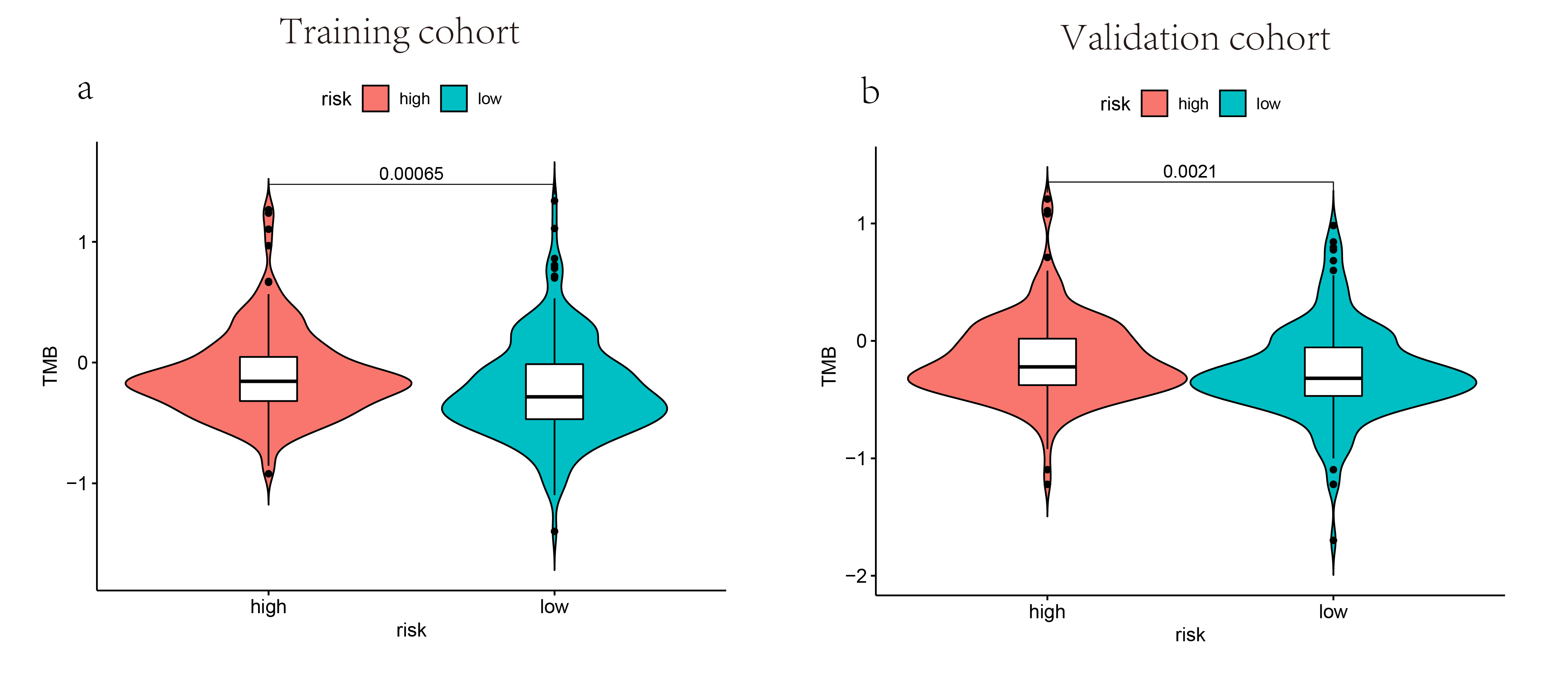

Supplement: Supplementary file 7 [file medi-102-e35978-s007.tif]
